# EBV and 1q Gains Affect Gene and miRNA Expression in Burkitt Lymphoma

**DOI:** 10.3390/v15091808

**Published:** 2023-08-25

**Authors:** Nuray Akyüz, Snjezana Janjetovic, Susanne Ghandili, Carsten Bokemeyer, Judith Dierlamm

**Affiliations:** Department of Oncology, Hematology and Bone Marrow Transplantation with Section Pneumology, University Clinic Hamburg-Eppendorf, 20251 Hamburg, Germany; n.akyuez@uke.de (N.A.); s.janjetovic@uke.de (S.J.); s.ghandili@uke.de (S.G.); cbokemeyer@uke.de (C.B.)

**Keywords:** Burkitt lymphoma, 1q gains, EBV, FISH, qPCR, miRNA

## Abstract

Abnormalities of the long arm of chromosome 1 (1q) represent the most frequent secondary chromosomal aberrations in Burkitt lymphoma (BL) and are observed almost exclusively in EBV-negative BL cell lines (BL-CLs). To verify chromosomal abnormalities, we cytogenetically investigated EBV-negative BL patient material, and to elucidate the 1q gain impact on gene expression, we performed qPCR with six 1q-resident genes and analyzed miRNA expression in BL-CLs. We observed 1q aberrations in the form of duplications, inverted duplications, isodicentric chromosome idic(1)(q10), and the accumulation of 1q12 breakpoints, and we assigned 1q21.2–q32 as a commonly gained region in EBV-negative BL patients. We detected *MCL1*, *ARNT*, *MLLT11*, *PDBXIP1*, and *FCRL5,* and 64 miRNAs, showing EBV- and 1q-gain-dependent dysregulation in BL-CLs. We observed *MCL1*, *MLLT11*, *PDBXIP1,* and 1q-resident miRNAs, hsa-miR-9, hsa-miR-9*, hsa-miR-92b, hsa-miR-181a, and hsa-miR-181b, showing copy-number-dependent upregulation in BL-CLs with 1q gains. *MLLT11*, hsa-miR-181a, hsa-miR-181b, and hsa-miR-183 showed exclusive 1q-gains-dependent and *FCRL5*, hsa-miR-21, hsa-miR-155, hsa-miR-155*, hsa-miR-221, and hsa-miR-222 showed exclusive EBV-dependent upregulation. We confirmed previous data, e.g., regarding the EBV dependence of hsa-miR-17-92 cluster members, and obtained detailed information considering 1q gains in EBV-negative and EBV-positive BL-CLs. Altogether, our data provide evidence for a non-random involvement of 1q gains in BL and contribute to enlightening and understanding the EBV-negative and EBV-positive BL pathogenesis.

## 1. Introduction

Abnormalities affecting the long arm of chromosome 1 (1q) are among the most common chromosomal alterations in human neoplasm and are known to frequently occur as a secondary change during disease progression [1,2,3,4]. In B-cell non-Hodgkin’s lymphomas (B-NHL), 1q rearrangements are observed at high frequency and are associated with a shortened median survival [2,5]. In Burkitt lymphoma (BL), structural abnormalities of 1q represent the most frequent secondary chromosomal aberrations and have been seen in 31% of all cytogenetically characterized cases [6].

BL is a highly aggressive B-NHL [7]. According to the clinical characteristics, endemic (predominantly in tropical Africa, Epstein–Barr virus-positive (EBV+)), sporadic (outside of tropical Africa, rarely EBV+), and HIV-associated BL forms are common [8]. Cytogenetically, BL is defined by the presence of the chromosomal translocation t(8;14)(q24;q32) or its variants t(2;8)(p12;q24) and t(8;22)(q24;q11) [6,9,10]. These translocations induce the overexpression of the oncogene *MYC* due to its juxtaposition to the immunoglobulin heavy-chain (*IGH*) locus on chromosome 14, or in their variants to the immunoglobulin light-chain loci kappa (*IGK*) and lambda (*IGL*) on chromosomes 2 and 22, respectively. In addition to *MYC* rearrangements, duplications involving the whole 1q arm or parts of it, whole 1q arm translocations, or jumping translocations involving 1q are widely reported in BL [11,12,13,14,15]. Duplications of the 1q21 region are found in up to 44% of BL cases [16]; however, the precise nature of cytogenetic aberrations leading to these molecular rearrangements in BL has so far not been analyzed in detail [6,16]. Moreover, being the most frequent secondary chromosomal change, the prognostic significance of 1q gains in BL is controversial. According to some authors, abnormalities involving 1q are associated with a poor prognosis and short survival in BL patients [2,5,17,18,19], whereas in some other reports, they do not seem to have any impact on the outcomes [20,21], underlining the importance of detailed investigations of these frequent but poorly understood cytogenetic alterations.

Micro-RNAs (miRNAs) are a group of endogenously encoded, short (~22 nucleotides), single-stranded, non-coding but functional RNA molecules that influence almost every cellular process, including development, differentiation, proliferation, and apoptosis, in various organisms [22,23,24,25]. The tightly regulated interaction between miRNAs and their target(s) in healthy cells is lost in cancer, and this dysregulation allows the expression of proteins with oncogenic properties, which seem to play significant roles not only in the initiation and development of cancer and the promotion of neoplastic transformation and tumorigenesis, but also in contributing to the classification and prognosis of different cancer entities [23,25,26,27,28,29,30]. Additionally, in BL, aberrant miRNA and mRNA expression profiles are observed [31,32], whereas differences in expression associated with 1q aberrations have not yet been reported.

In our previous study, we cytogenetically analyzed well-established BL cell lines (BL-CLs) and observed aberrations involving 1q as the most frequent structural change, as well as an accumulation of 1q12 breaks [33]. Furthermore, we found a correlation between the absence of EBV and the presence of 1q gains, and defined 1q21.2–q22 as a minimal commonly gained region, when duplications and partial trisomies were considered together [33]. In order to prove this correlation on primary material, we cytogenetically analyzed three EBV-negative (EBV−) patient samples. Furthermore, we investigated EBV status- and 1q-gain-dependent gene and miRNA expression in BL-CLs in order to find specific patterns for EBV+ and EBV− BL. Altogether, our results indicate an accumulation of 1q aberrations in BL and a non-random involvement of 1q aberrations in EBV− and EBV+ BL by affecting mRNA and miRNA expression.

## 2. Materials and Methods

### 2.1. BL Patient Samples and Cell Lines

Three EBV− BL patient samples were selected from the archival methanol-acetic-acid-fixed cytogenetic preparations from the University Medical Center, Hamburg-Eppendorf, Hamburg, Germany (n = 1, patient-1 (male), Table 1), and the Center for Human Genetics of the University of Leuven, Leuven, Belgium (n = 2, patient-2 (male) and -3 (female), Table 1). All BL patients were studied at the time of primary diagnosis, were classified according to the WHO classification [7], and showed the typical histological features of BL. The G-banding karyotypes of the patients at initial diagnosis are summarized in Table 1. Our study was performed in accordance with the Declaration of Helsinki from 1975 and informed consent was obtained from all patients.

Since fresh, frozen primary material from patients was not available, gene and miRNA expression were investigated using BL-CLs. The main characteristics of the used BL-CLs are summarized in Table 2, and their updated karyotypes are published in our previous report [33].

### 2.2. Multicolor Fluorescence In Situ Hybridization and Probe Selection for Metaphase FISH Mapping of 1q Abnormalities

Multicolor fluorescence in situ hybridization (mFISH) was performed using the 24Xcyte color kit for human chromosomes (MetaSystems, Altlussheim, Germany) according to the supplier’s recommendations. Dual-color metaphase FISH was performed according to our previous publication [33]. For the metaphase FISH 37, partially overlapping BAC and PAC clones spanning the whole 1q-arm were selected from information archived (on January 2005) by the National Center for Biotechnology Information (NCBI) (http://www.ncbi.nlm.nih.gov/; 1 January 2005) and obtained from the RPCI-1, -3, -4 and -11 libraries (Roswell Park Cancer Institute, Buffalo, NY, USA) (Supplemental Table S1). Details of mFISH and FISH are summarized in the supplemental materials and methods sections. Karyotypes were described according to the International System for Human Cytogenetic Nomenclature (ISCN, 2020) [34] and revised using the online analysis webpage CyDAS (http://www.cydas.org/, 1 July 2023).

### 2.3. RNA Preparation, Reverse Transcription and Real-Time qPCR Analysis

Total RNA was isolated from 37 BL-CLs (Table 2) using the RNeasy Mini Kit (QIAGEN, Hilden, Germany) and cDNA synthesis was performed using SuperScript™ II RT (Invitrogen, Darmstadt, Germany) according to the manufacturer’s instructions. Real-time qPCR was performed on a Light Cycler Instrument (Roche, Basel, Switzerland) using the QuantiFast SYBR Green PCR Kit (QIAGEN). Primers for HMBS were obtained from Eurofins (Ebersberg, Germany). The QuantiTect Primer Assay from QIAGEN (Supplemental Table S2) was used for *BCL9*, *MCL1*, *ARNT*, *MLLT11*, *PBXIP1* and *FCRL5* amplification. The qPCR data were analyzed according to the comparative CT method (Applied Biosystems, Darmstadt, Germany) using the endogenous reference *HMBS* as a normalization factor. Normalized mRNA levels detected in BL-28, i.e., an EBV− BL-CL without 1q gains, were averaged and set to 1 and the amounts measured in the remaining BL-CLs were calculated relative to those levels. Each sample was measured in two independent qPCR experiments prepared as duplicates.

### 2.4. Micro RNA Analysis

MiRNA was isolated from 12 BL-CLs (Table 2, dark grey highlighted) during two independent days using the miRNeasy mini kit (QIAGEN) according to the manufacturer’s instructions. MiRNA expression analysis using miRCURY Hy3/Hy5 dual-color labeling and hybridization with the miRCURY™ LNA Array (EXIQON, Vedbaek, Denmark) was performed by the company EXIQON. The clustering was performed using log2(Hy3/Hy5) ratios, which passed the filtering criteria on variation across sample groups using a two-tailed *t*-test; a *p*-value < 0.05 and only miRNA with values showing a significant difference were considered.

## 3. Results

### 3.1. EBV-Negative BL Accumulate 1q Gains

Gains of 1q arm were mainly observed in EBV− BL-CLs [33,35,36]. We observed a correlation between the absence of EBV and gain of 1q in BL-CLs [33]. To verify this correlation on primary material, we analyzed the samples from three EBV− BL patients. To determine the exact position of the breakpoints and to uncover cryptic 1q rearrangements, we performed classical G-banding, mFISH and FISH with 37 specific clones covering the whole 1q arm (Supplemental Table S1). As expected, the characteristic *MYC* rearrangement for BL involving 8q24 was present in all investigated patient samples detected as t(8;14)(q24;q32) (Table 1). The gain of 1q-arm was observed in all three patient samples; in contrast, balanced translocations involving 1q, loss of 1q or rearrangements involving 1p were not observed.

**Figure 1:**
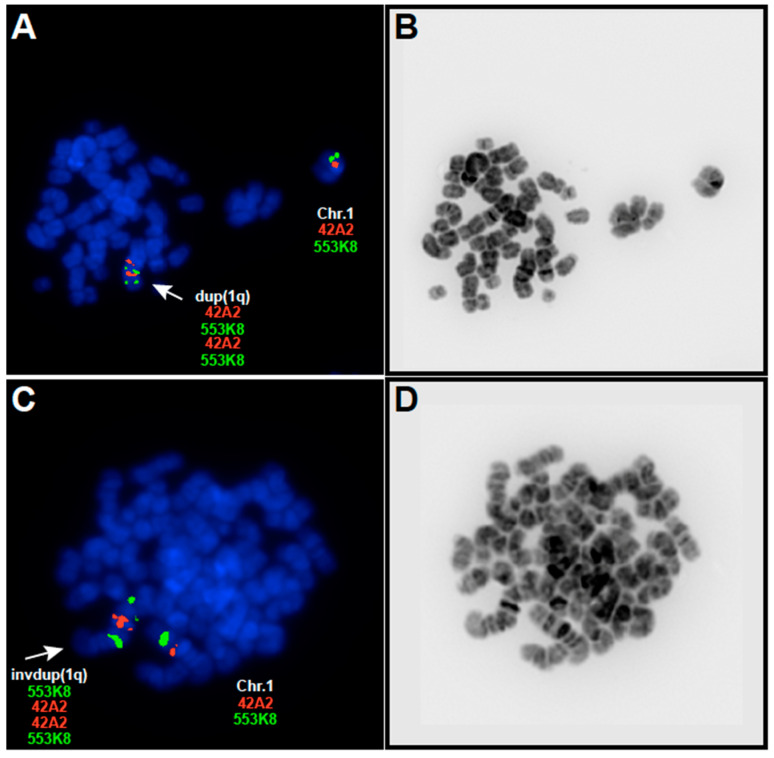

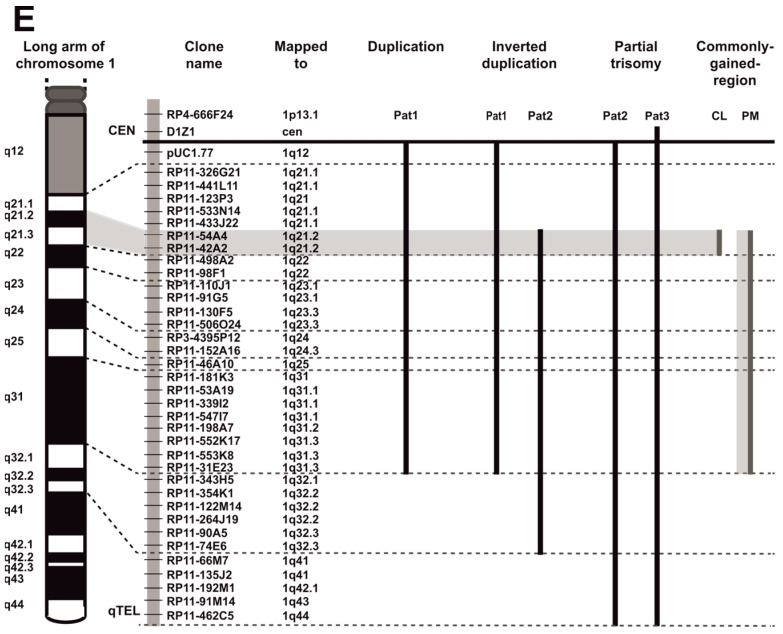
Analysis of the 1q abnormalities using dual-color metaphase FISH. Representative images showing 1q duplication (**A**) and inverted duplication (**C**) in patient-1 using the BAC clones RP11-553K8 (green) and RP11-42A2 (red). The abnormal chromosomes are indicated by arrowheads. In (**A**,**C**), representative dual color FISH images are shown and in (**B**,**D**), the corresponding G-banding images are shown (**E**) Summary of the 1q aberrations achieved by FISH on primary material. The BAC clones selected for this study and their cytogenetic positions are indicated on the right side of the 1q ideogram. The vertical lines indicate the overrepresented chromosomal region as assessed by FISH and correspond to the following patients (from left to right); duplications: patient-1; inverted duplications: patients-1 and -2; partial trisomies: patients-2 and -3. The minimal commonly gained-region 1q21.2–q22 delineated in our previous report using BL-CLs [33] is shaded in grey and indicated with CL (cell lines), and the commonly gained region 1q21.2–q32 delineated in our present study using primary material is indicated with PM (primary material). Pat1–3: Patient-1–3.

In samples of patient-1 and -2, we found intra-chromosomal duplication of 1q and interestingly, they uncovered concomitant inversions of the duplicated regions. Moreover, the sample from patient-1 displayed the concomitant presence of two cytogenetic clones with a duplication and an inverted duplication of the same 1q region (Figure 1, Table 1). In the sample of patient-2, we observed several sub-clones characterized by distinct and specific chromosome 1 rearrangements including the presence of partial trisomy of 1q presented as a translocation of 1q (region 1q12–q44) to the recurring partner at 7p22 on the der(7) and inverted duplication of 1q41–q21.2 (Figure 1E, Table 1). In the sample of patient-3, we observed, with all the applied 1q probes, three hybridization signals with a breakpoint at 1q10 indicating the presence of an isodicentric chromosome 1 idic(1)(q10) (Figure 1E, Table 1).

Considering the duplications and inverted duplications detected in the samples from patient-1 and in two clones of patient-2, the proximal border of 1q gains was flanked by the pericentric heterochromatic region 1q12 and 1q21.2 and the distal border by 1q32.1 and 1q41 (Figure 1E, Table 1). This clustering indicates the region 1q21.2–1q32.1 as a commonly gained region in the primary material.

Taken together, we observed in all EBV− BL patient samples 1q gains as the most prominent structural aberration in the form of duplications and inverted duplications and accumulation of 1q12 breaks. We assigned 1q21.2–q32.1 as a commonly gained region for the primary material and detected an isodicentric chromosome 1 idic(1)(q10) in BL.

### 3.2. EBV and 1q Gains Affect the Expression of Genes Localized at 1q21.2–q22

**Figure 2:**
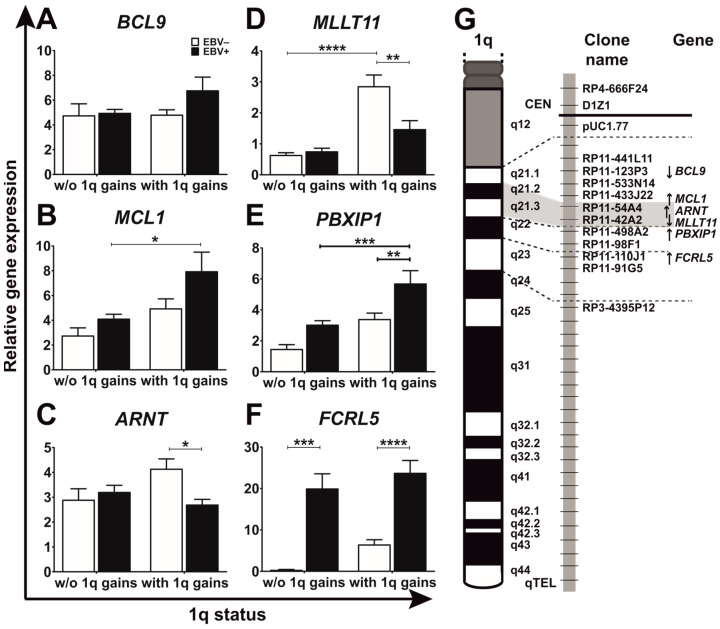
Differential expression of six genes located at 1q21.2–q22 in BL-CLs. The figure shows the expression level of *BCL9* (**A**), *MCL1* (**B**), *ARNT* (**C**), *MLLT11* (**D**), *PBXIP1* (**E**) and *FCRL5* (**F**) relative to their mRNA level detected in the BL-28, an EBV− BL cell line without 1q aberrations. The localization of the investigated genes, their transcriptional direction (indicated with an arrow) and the BAC clones covering their position are highlighted on the 1q ideogram (**G**). Expression levels in EBV-negative (EBV−) BL-CLs are shown as empty bars and in EBV-positive (EBV+) BL-CLs as black bars. Note that 4 EBV− and 12 EBV+ BL-CLs without (w/o) 1q gains and 13 EBV− and 8 EBV+ BL-CLs with 1q gains were investigated in this study. Each sample was measured in two independent qPCR experiments prepared as duplicates. *: *p* < 0.05; **: *p* < 0.01; ***: *p* < 0.001; ****: *p* < 0.0001 (two-way ANOVA followed by Tukey’s multiple comparisons test).

**Figure 3:**
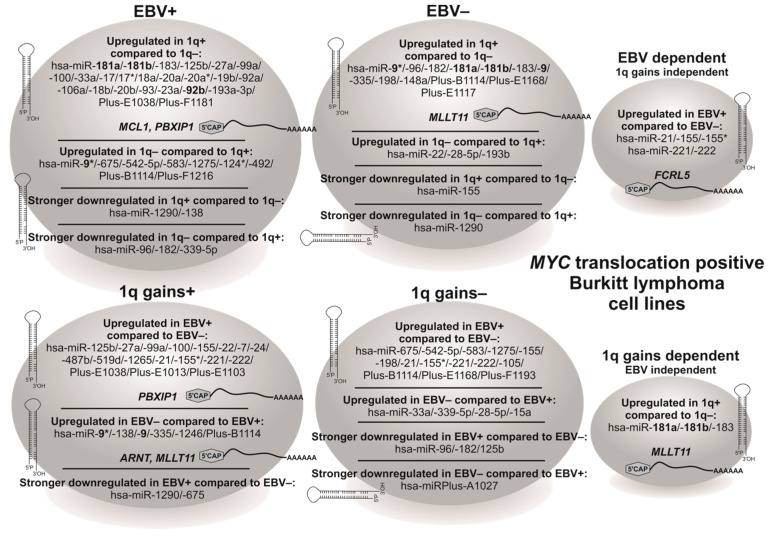
Summary of the expression profiles of the significantly dysregulated mRNAs and miRNAs in MYC translocation positive BL-CLs depending on their EBV and 1q gain status. The 1q resident miRNAs hsa-miR-9, hsa-miR-9*, hsa-miR-92b, hsa-miR-181a and hsa-miR181b are highlighted in bold letters. The significantly dysregulated mRNAs are indicated in bold and italic letters. The drawing of a hairpin structure indicates miRNA and curved line with a 5′-Cap and 3′-poly-A tail indicates mRNA.

Using BL-CLs, we defined 1q21.2–q22 as minimal commonly gained region, which was flanked in its centromeric margin by the clone RP11-54A4 (*MCL1*) and telomeric margin by RP11-42A2 (telomeric to *MLLT11*) (Figure 2G, Supplemental Table S1) [33]. In order to analyze whether EBV and/or 1q gains affect the expression of genes located within or flanking this minimal commonly gained region, we used 37 BL-CLs (Table 2) and performed qPCR with six genes, namely *BCL9*, *MCL1*, *ARNT*, *MLLT11*, *PBXIP1* and *FCRL5* (Figure 2 and Figure 3), which are reported to be involved in B-lymphocyte development and malignancy.

We observed similar *BCL9* expression in all investigated groups with a slight increase in EBV+ BL-CLs with 1q gains (Figure 2A). Compared to EBV+ BL-CLs without 1q gains, the *MCL1* and *PBXIP1* levels measured in EBV+ BL-CLs with 1q gains were 2-fold higher (Figure 2B,E and Figure 3). We detected 1.5-fold higher *ARNT* expression in EBV− BL-CLs with 1q gains compared to EBV+ BL-CLs with 1q gains (Figure 2C and Figure 3). The *MLLT11* levels in EBV− BL-CLs with 1q gains were 4-fold higher compared to EBV− BL-CLs without 1q gains and 2-fold higher compared to EBV+ BL-CLs with 1q gains (Figure 2D and Figure 3). Compared to EBV− BL-CL, the *FCRL5* amounts in EBV+ BL-CLs with and without 1q gains were 4-fold and 57-fold higher, respectively (Figure 2F and Figure 3).

Taken together, *MCL1*, *ARNT*, *MLLT11*, *PBXIP1* and *FCRL5*, genes located within the minimal commonly gained region 1q21.2–q22 as assigned in BL-CLs [33], showed EBV- and 1q-gains-dependent expression differences. We observed *FCRL5* upregulated in EBV+ BL-CLs, *MCL1* and *PBXIP1* in EBV+ BL-CLs with 1q gains and *MLLT11* and *ARNT* in EBV− BL-CLs with 1q gains.

### 3.3. BL Cell Lines Show EBV- and 1q-Gains-Dependent Specific miRNA Expression Patterns

**Figure 4:**
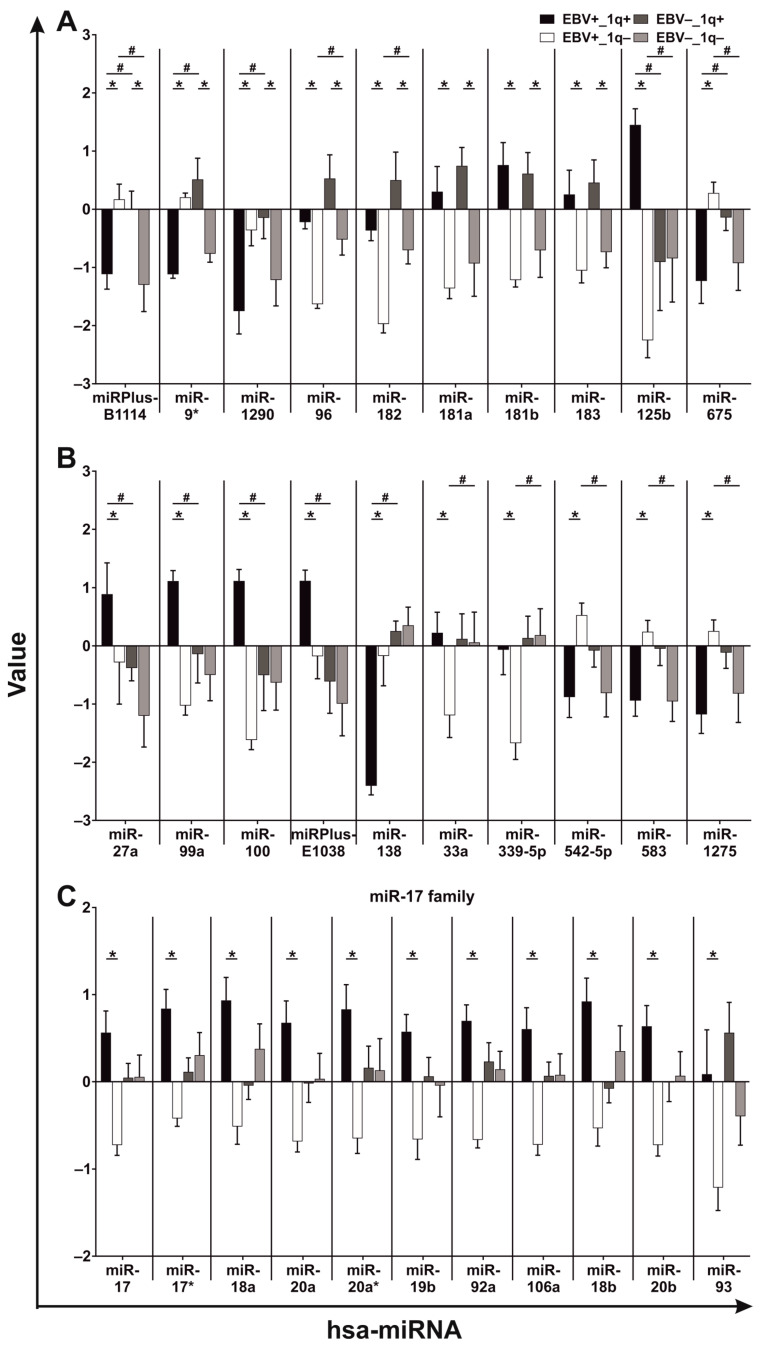

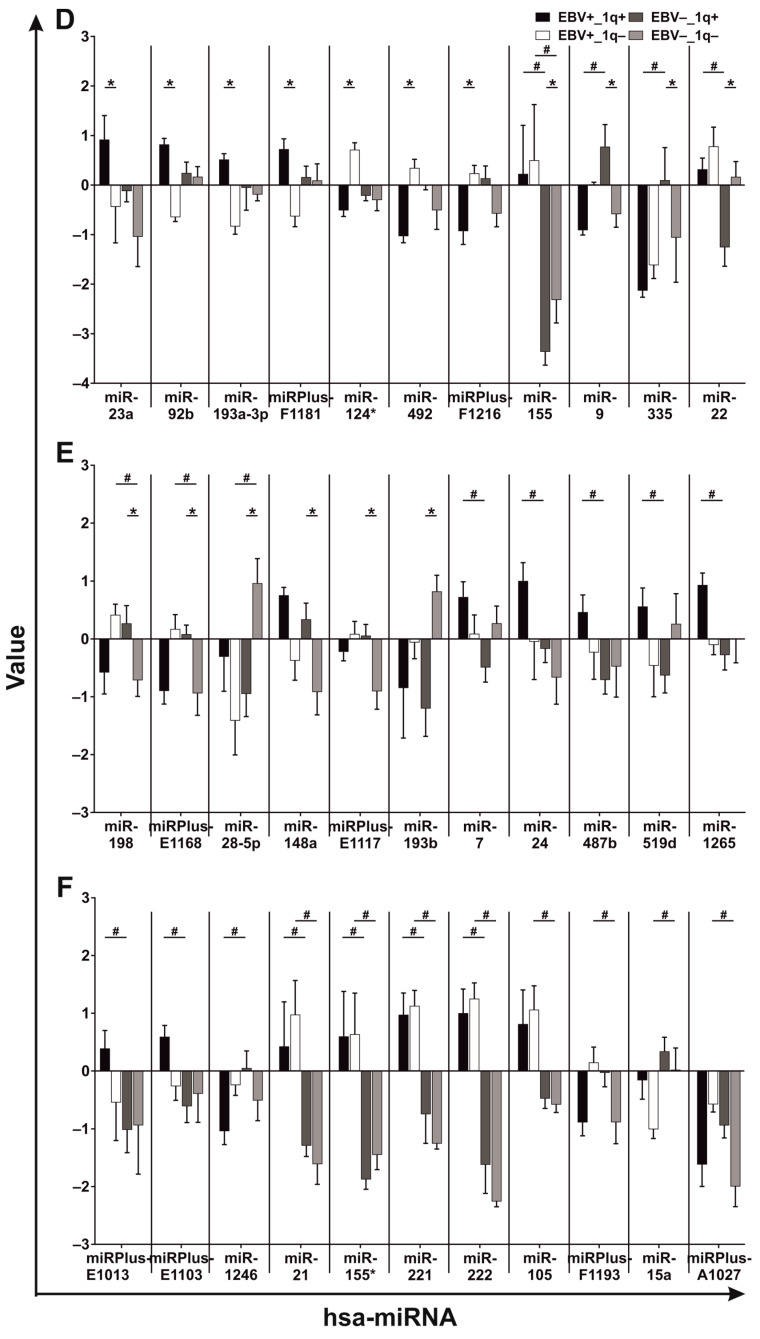
EBV- and 1q-gains-dependent miRNA expression in BL-CLs. Expression profiles of 64 significantly dysregulated miRNAs in BL-CLs depending on their EBV and 1q gain status are shown as diagrams (**A**–**F**). All miRNAs are numbered according to their appearance in this figure indicated under their names. Value: log2 normalized Hy3/Hy5 ratios. Statistical analysis: two-way ANOVA followed by Tukey’s multiple comparisons test, * indicates the comparison between the BL-CL groups with 1q gains (1q+) vs. without 1q gains (1q−) (*p* < 0.05) and # indicates the comparison between the BL-CL groups EBV-positive (EBV+) vs. EBV-negative (EBV−) (*p* < 0.05).

Aberrant miRNA and mRNA expression profiles were detected in different cancer entities and MYC was shown to upregulate, among others, the oncomiR miR-17-92 cluster [31,32,37], whereas an association to the cytogenetic status, especially 1q gains, was not reported. Additionally, EBV is associated with BL malignancy, whereas the characteristics of EBV− BL have not been fully investigated Thus, in order to analyze whether EBV or 1q gains affect miRNA expression, we investigated 12 BL-CLs (Table 2) and detected 64 differentially expressed miRNAs (Figure 3 and Figure 4, Supplemental Tables S3 and S4). The miRNA and qPCR results from the previous subsection are summarized in Figure 3.

We observed 38 miRNAs that were dependent on 1q gains and significantly dysregulated in EBV+ and 18 in EBV− BL-CLs (Figure 3 and Figure 4, Supplemental Table S3). In contrast, 26 miRNAs were EBV-dependent and significantly dysregulated in BL-CLs with 1q gains and 22 in those without 1q gains (Figure 3 and Figure 4, Supplemental Table S4). Three miRNAs, namely hsa-miR-181a, hsa-miR-181b and hsa-miR-183, are independent of their EBV status upregulated in BL-CLs with 1q gains (Figure 4A, no. #6-#8). In addition, hsa-miR-125b, hsa-miR-27a, hsa-miR-99a, hsa-miR-100 and hsa-miRPlus-E1038 were upregulated and hsa-miR-675 and hsa-miR-138 were downregulated in EBV+ BL-CLs with 1q gains compared to EBV+ BL-CLs without 1q gains and EBV− BL-CLs with 1q gains (Figure 4A,B, no. #9-#15). The hsa-miR-17-92 cluster members (hsa-miR-17, hsa-miR-17*, hsa-miR-18a, hsa-miR-20a, hsa-miR-19b, hsa-miR-92a) and their paralogues (hsa-miR-18b, hsa-miR-20b, hsa-miR-106a and hsa-miR-93) were EBV-dependent and upregulated in BL-CLs with 1q gains (Figure 4C, no. #21-#31). Moreover, hsa-miR-21, hsa-miR-155/-155*, hsa-miR-221 and hsa-miR-222 were EBV-dependent and upregulated in EBV+ BL-CLs, both with and without 1q gains, whereby hsa-miR-155 was more downregulated in EBV− BL-CLs with 1q gains (Figure 4D,F, no. #39,#57-#60). Additionally, hsa-miR-9, hsa-miR-148a, hsa-miR-198, hsa-miR-335, hsa-miRPlus-E1117 and hsa-miRPlus-E1168 were upregulated and has-miR-22, hsa-miR-28-5p and hsa-miR-193b were downregulated in EBV− BL-CLs with 1q gains compared to EBV− BL-CLs without 1q gains (Figure 4D,E, no. #40-#48). We also observed both mature and passenger strands in five miRNAs: hsa-miR-9/-9*, hsa-miR-17/-17*, hsa-miR-20a/-20a* and hsa-miR-155/-155*, which were dysregulated in a similar way, whereas only the passenger strand for hsa-miR-124* was significantly dysregulated in the investigated BL-CLs (Figure 4A,C,D,F, no. #2,#40,#21,#22,#24,#25,#39,#58,#36). Interestingly, hsa-miR-1290 was downregulated in all investigated BL-CLs and showed a stronger downregulation in both EBV+ BL-CLs with 1q gains and EBV− BL-CLs without 1q gains (Figure 4A, no. #3).

It is also noteworthy that 5 of the 64 significantly dysregulated miRNAs were localized on the long arm of chromosome 1, namely hsa-miR-181a and hsa-miR-181b at 1q32.1, hsa-miR-9, hsa-miR-9* and hsa-miR-92b at 1q22, and all of them were upregulated in BL-CLs with 1q gains (Figure 4A,D, no. #6,#7,#2,#40,#33). While hsa-miR-92b was upregulated in EBV+ BL-CLs with 1q gains, hsa-miR-181a and hsa-miR-181b were both upregulated in EBV+ and EBV− BL-CLs with 1q gains. In contrast, hsa-miR-9 and hsa-miR-9* were upregulated in EBV− BL-CLs with 1q gains. Additionally, hsa-miR-9* was downregulated in EBV+ BL-CLs with 1q gains and upregulated in those without 1q gains (Figure 4A,D, no. #6,#7,#2,#40,#33, Supplemental Tables S3 and S4).

Taken together, we observed 64 miRNAs that were EBV- and 1q-gains-dependent dysregulated in BL-CLs. Interestingly, five miRNAs, namely hsa-miR-181a, hsa-miR-181b, hsa-miR-9, hsa-miR-9* and hsa-miR-92b localized to 1q, were upregulated in BL-CLs with 1q gains. Additionally, hsa-miR-181a, hsa-miR-181b and hsa-miR-183 were upregulated in BL-CLs with 1q gains independent of their EBV status and hsa-miR-21, hsa-miR-155, hsa-miR-155*, hsa-miR-221, and hsa-miR-222 were upregulated in EBV+ BL-CLs independent of their 1q gains status.

## 4. Discussion

Chromosomal rearrangements affecting 1q are the most common secondary abnormalities observed in BL [6,33]. In our previous report, we described a correlation between the absence of EBV and gain of 1q in BL-CLs, and verified 1q21.2–q22 as a minimal commonly gained region [33]. To determine whether primary material shows a similar correlation in this study, we carried out a cytogenetic investigation on three EBV− BL patient samples. Additionally, we performed miRNA and gene expression analyses in BL-CLs to elucidate specific patterns depending on 1q gains and EBV characterizing BL in detail.

In all EBV− BL patient samples, we observed 1q gains in the form of intra-chromosomal duplication of 1q, translocation of the whole 1q arm and isodicentric chromosome 1 idic(1)(q10), resulting in partial tetrasomy of the 1q arm. While isodicentric chromosome idic(1)(p12) [12] and isochromosome i(1)(q10) [13] have already been reported, this is the first report describing supernumerary idic(1)(q10) in BL. Isochromosomes implicate the presence of a tetrasomy of the involved chromosome arm and imply the dysregulated expression of (proto-)oncogenes encoded by the affected chromosome arm [12,13]. Moreover, patient-1 displayed the concomitant presence of two cytogenetic clones bearing a duplication and an inverted duplication of the same 1q region. This is, to the best of our knowledge, the first report that shows that chromosomal duplications and inversions are temporarily separated events and that, at least in this case, the inversion followed the duplication of the 1q material.

The long arm of chromosome 1 is prone to breaks in cancer and 1q gains involve large regions covering nearly the whole 1q arm [2,33,38]. In our study, the proximal border of the duplicated 1q region in EBV− patients was mapped to the pericentric heterochromatic region 1q12 and 1q21.2 and the telomeric breakpoints were clustered in chromosomal band 1q32 and 1qter, indicating 1q21–2-1q32 as the minimal commonly gained region, which covers the minimal commonly gained region 1q21.2–q22 assigned in BL-CLs [33]. In contrast to the heterogeneous distribution of the distal breakpoints, we observed an accumulation of 1q gains with a centromeric breakpoint at the constitutive heterochromatin band 1q12 in both BL-CLs [33] and primary material indicating 1q12 as a non-random target of rearrangements in BL. The recurrence of breakpoints involving the pericentric heterochromatin band 1q12 was also observed in other NHL entities [2,3,39,40,41,42], pointing to 1q12 as an instability region that may contribute to lymphomagenesis. The juxtaposition of euchromatin regions to the constitutive heterochromatin might lead to the repression of gene(s) adjacent to the breakpoint. Thus, an excess of heterochromatin may result in the silencing of target genes by the formation of aberrant transcriptionally silent domains [41,43]. Heterochromatin region 1q12 tends to be situated close to the membrane of the nucleus and this perinuclear localization of heterochromatin is associated with transcriptional silencing [43,44]. Nevertheless, the peripheral localization of 1q12 heterochromatin is disrupted in lymphoma cells harboring 1q12 rearrangements; therefore, the information required for gene silencing might be lost in tumor cells [43]. Hence, it is highly suggestive to think that the loss of the silencing mechanisms of target genes in BL with 1q12 alterations could be potentiated by the gain of 1q regions harboring those target genes. In fact, the chromosomal pericentromeric regions are often composed of duplicated low copy repeat sequences, suggesting a link between duplications and genomic instability at the pericentric heterochromatin regions of chromosomes [45].

Gains of the 1q-arm are mainly observed in EBV− BL-CLs [33,35,36], and authors have hypothesized that genetic information similar to that included in viral genome exists on 1q [36]. A gene dosage effect resulting in higher copy numbers of gene(s), potentially relevant for proliferation, has been discussed to explain the biological consequences underlying chromosomal trisomies [46]. Additionally, 1q-resident genes *MDM4* and hsa-miR-181b were overexpressed following 1q gains in BL and were discussed as candidate disease drivers [47,48,49]. In this regard, commonly overrepresented regions and partial trisomies are of particular significance and can delineate subregions bearing genes that are important for pathogenicity. Therefore, we assumed that candidate genes important for BL malignancy might exist in the minimal commonly gained region 1q21.2–q22 delineated in BL-CLs [33]. This region is flanked in its centromeric margin by the clone RP11-54A4 (covers *MCL1* gene) and telomeric margin by RP11-42A2 (telomeric to *MLLT11*). Thus, we decided to investigate the expression of *BCL9*, *MCL1*, *ARNT*, *MLLT11*, *PBXIP1* and *FCRL5* genes known to be involved in B-cell development and malignancies and localized or adjacent to the minimal commonly gained region 1q21.2–q22. *BCL9* oncogene, a member of the Wnt signaling pathway, are aberrantly expressed in human multiple myeloma (MM) and colon carcinoma [50] and overexpressed following t(1;14)(q21;q32)/BCL9-IGH in pre-B-cell acute lymphoblastic leukemia (ALL) [51]. *BCL9* is localized at 1q21.2 but above the minimal commonly gained region [33]. Accordingly, we observed neither EBV- nor 1q-gains-dependent differences in *BCL9* expression. *MLLT11* is involved in t(1;11)(q21;q23) and high MLLT11 expression, which is detected in pediatric acute myeloid leukemia (AML), is associated with poor outcomes [52]. MCL1 is a member of the BCL2 family of anti-apoptotic, prosurvival proteins and is frequently overexpressed in high-grade NHL lymphomas and MM [53,54,55]. Furthermore, MYC-driven lymphomas are highly dependent on MCL1 for survival [56]. In addition, MCL1 is induced after the EBV transformation of B-cells [57,58]. FCRL5, a B-cell membrane protein involved in t(1;14)(q21;q32), is upregulated in BL-CLs with 1q21 abnormalities and is induced by EBV [59,60]. PBXIP1, involved in organogenesis and tumorigenesis, is overexpressed in various tumors [61,62]. ARNT is involved in xenobiotic metabolism and hypoxia responses and stimulates angiogenesis in different malignancies [63]. Increased ARNT expression is associated with drug resistance in high-risk MM [64] and t(1;12)(q21;p13)/TEL(ETV6)-ARNT, resulting in an impaired transcriptional response and hematopoietic cellular differentiation, is associated with AML [65,66]. The involvement of *BPXIP1* or *ARNT* in BL or an association with EBV has not yet been reported. Here, we confirmed the EBV dependence of *MCL1* and *FCRL5*. Our results show that *MCL1* and *PBXIP1* increased in 1q-gained EBV+ and *MLLT11* and *ARNT* increased in EBV− BL-CLs with 1q gains. It indicates a dosage-dependent dysregulation of genes located at the gained 1q bands in BL, strengthening its role in the pathogenesis of BL with 1q gains.

One of the mechanisms used to control gene expression includes post-transcriptional regulation by miRNAs. Expression of miRNAs in BL was investigated in different experimental approaches. However, a relation to EBV and 1q gains has not yet been reported. Here, we investigated miRNA expression in MYC-Ig translocation-positive BL-CLs and observed 64 miRNAs that were EBV- and 1q-gains-dependent dysregulated in BL-CLs: 21 of them were known to be dysregulated in BL, 10 miRNAs were not delineated yet and the remaining 33, were described for the first time in this paper to be dysregulated in BL. However, these 33 miRNAs were found to be dysregulated in other cancer entities (s. references in Supplemental Table S5).

The most extensively investigated oncomiR hsa-miR-17-92 cluster, involved in B-cell development, is dysregulated in different cancer entities and EBV-infected B-cells and is known to be under the transcriptional control of MYC. It is also differentially regulated in MYC-driven cancers, among others in BL [25,31,32,37,67,68,69,70]. We confirmed its EBV dependence and observed the hsa-miR-17-92 cluster members and their paralogues upregulated in EBV+ BL-CLs with 1q gains, indicating an additive regulatory effect caused by 1q gains in the presence of EBV. Additionally, MYC was shown to stimulate hsa-miR-9 and repress hsa-miR-15a, hsa-miR-22, hsa-miR-23a, hsa-miR-100/let-7a-2/hsa-miR-125b-1 clusters and hsa-miR-28. Altogether, they are involved in hematopoiesis, proliferation, apoptosis, cell cycle control and survival [68,71,72,73,74]. Our data indicate that these MYC-regulated miRNAs are additionally affected by EBV and 1q gains in BL and, in case of hsa-miR-28-5p, a possible regulation independent of EBV and 1q gains.

Dysregulation of some miRNAs was directly linked to frequent chromosomal alterations involving their chromosomal loci and increased hsa-miR-9 expression was associated with the presence of 1q gains [75]. In this regard, 5 of the 64 miRNAs observed as dysregulated in our study are localized to 1q, namely hsa-miR-9, hsa-miR-9* and hsa-miR-92b at 1q22 and hsa-miR-181a and hsa-miR-181b at 1q32.1. Thus, the upregulation of these 1q resident miRNAs and four 1q resident genes (*MCL1*, *PBXIP1*, *MLLT11* and *ARNT*) in BL-CLs with 1q gains confirms previous results and supports the assumption of a copy-number effect associated with these chromosomal aberrations [75].

DNA methyltransferases, responsible for the methylation of the CpG islands, are involved in gene expression and are repressed by hsa-miR-148a [72]. Hsa-miR-1275, a key regulator of apoptosis, autophagy and different signaling pathways, is involved in the development of different cancer types [76]. Hsa-miR-222/-221 clusters, involved in cell cycle control, are frequently upregulated in cancer and are induced by EBV [77,78,79]. Additionally, a strong relation between EBV presence and hsa-miR-21 and hsa-miR-155 expression was confirmed [80,81]. Both miRNAs are involved in hematopoiesis and are upregulated in cancer [25]. In another study, hsa-miR-155 was upregulated and the hsa-miR-183-96-182 cluster was downregulated in EBV+ BL-CLs [82]. Compared to GC B-cells, hsa-miR-182-5p, hsa-miR-183-5p and hsa-miR-148a-3p were upregulated and hsa-miR-28-5p, hsa-miR-222-3p, hsa-miR-221-3p and hsa-miR-1275 were downregulated in endemic BL tumor cells [31]. We confirmed the EBV dependence of hsa-miR-21, hsa-miR-155/-155*, hsa-miR-183-96-182 clusters and hsa-miR-222/-221 clusters, whereas our data indicate a different control mechanism involving hsa-miR-148a, the hsa-miR-183-96-182 family member and hsa-miR-1275 associated with 1q gains in EBV− and EBV+ BL. A different cellular origin within the BL subtypes is hypothesized. While EBV+ BL was assumed to originate from late-GC or memory (post-GC) B-cells, EBV− (mainly sporadic) BL appears to develop from early centroblasts [83,84]. Thus, the use of different experimental designs, cancer entities and control cell lines/tissues may cause the above-mentioned expression differences regarding these miRNAs observed in our study and previous studies.

In summary, we were able to confirm the already-known EBV dependence of some miRNAs in BL-CLs and achieved additional information taking 1q gains and EBV status into account. We observed five 1q resident miRNAs (hsa-miR-9, hsa-miR-9*, hsa-miR-92b, hsa-miR-181a and hsa-miR-181b) that were upregulated in BL-CLs with 1q gains, three miRNAs (hsa-miR-181a, hsa-miR-181b and hsa-miR-183) upregulated in BL-CLs with 1q gains, independent of their EBV status, and five miRNAs (hsa-miR-21, hsa-miR-155, hsa-miR-155*, hsa-miR-221, and hsa-miR-222) upregulated in EBV+ BL-CLs, independent of their 1q gain status. Altogether, our data indicate EBV- and 1q-gains- dependent concerted action of 64 miRNAs in BL-CLs, which may contribute to the EBV− and EBV+ BL malignancies. The miRNAs, here observed as dysregulated, are involved in proliferation, invasion, migration, cell cycle control, apoptosis and epigenetic regulation like histone-modifications or promoter methylation. Their dysregulation in cancer provides tumor cells with an attractive environment, benefits and unhampered growth. It will be challenging to elucidate the functional relevance of the individual miRNAs observed as dysregulated in our study.

## 5. Conclusions

We cytogenetically investigated EBV− BL primary material and analyzed EBV− and EBV+ BL-CLs in order to find 1q-gains-dependent differences in gene and miRNA expression. We observed an accumulation of 1q gains in EBV− BL cases and EBV- and 1q-gains-dependent specific gene and miRNA expression patterns involving five 1q resident mRNAs and 64 miRNAs in BL-CLs. Altogether, our data indicate a non-random involvement of 1q gains in BL and contribute to a better understanding of EBV− and EBV+ BL pathogenesis. Future investigations should be focused on primary material to confirm the effects of 1q gains in gene and miRNA expression, as well as on the influence of the condensed heterochromatin 1q12 band in the pathogenesis of BL. These would enlighten the molecular genetic differences in EBV− and EBV+ BL and provide new markers to develop novel, precise and personalized therapeutic approaches to improve diagnosis and prognosis of BL.

## Figures and Tables

**Table 1:** Cytogenetic features of patients analyzed in this study using G-banding, mFISH and FISH.

Patient	G-Banding Karyotype	Partial mFISH Karyotype	Partial FISH Karyotype ^a^
Pat1	44-45,XY,dup(1)(q22q31),-3,-4, t(8;14)(q24;q32),del(11)(q23),+mar[cp25]	45,XY,dup(1)(q22q31),t(8;14)(q24;q32)	I. dup(1)(q12q32.1)/ pUC1.77 to RP11-31E23 (*PTPRC*) II. dup(1)(q32.1q12)/ RP11-31E23 (*PTPRC*) to pUC1.77
Pat2	45-47,XY,dup(1)(q21q44)[4], der(6)t(1;6)(q21;q27)[1],del(6)(q22q25)[1], der(7)t(1;7)(q21;p22)[3], t(8;14)(q24;q32)[cp9],+11 [cp9]	I.46,XY,der(7)t(1;7)(q;p),t(8;14)(q24;q32) II.43,XY,dup(1q),t(8;14)(q24;q32)	I. der(7)t(1;7)(q12;p22)/ pUC1.77 to 1qter II. dup(1)(q41q21.2)/ RP11-74E6 (*PTPN14*) to RP11-54A4 (*MCL1*)
Pat3	47,XX,+idic(1)(q10),t(8;14)(q24;q32), del(10)(p14),der(14)t(8;14)[10]	47,XX,+idic(1)(q10),t(8;14)(q24;q32), der(14)t(8;14)	idic(1)(q10)/ centromere 1 to 1qter

^a^ Used FISH probes are indicated after the slash and are summarized in Supplemental Table S2. Pat1–3: Patient-1–3.

**Table 2:** Characteristics of the Burkitt lymphoma cell lines used in this study.

No.	BL Cell Line ^a^	EBV Status	1q Gain Status	MYC Translocation	Sex	Age	Order No.	Purchased
1	BL-28	−	−	t(8;14)(q24;q32)	m	19	−	Prof. G. Klein ^b^
2	BLUE-1	−	−	t(8;14)(q24;q32)	m	29	ACC 594	DSMZ ^c^
3	DG-75	−	−	t(8;14)(q24;q32)	m	10	ACC 83	DSMZ ^c^
4	Ramos	−	−	t(8;14)(q24;q32)	m	3	ACC 603	DSMZ ^c^
5	BL-2	−	par-tri; del	t(8;22)(q24;q11)	m	7	−	Prof. G. Klein ^b^
6	BL-92	−	par-tri	t(8;14)(q24;q32)	m	14	IARC1503	IARC ^d^
7	ST-486	−	par-tri	t(8;14)(q24;q32)	n.a.	n.a.	−	Prof. G. Klein ^b^
8	BL-41	−	inv-dup; par-tri	t(8;14)(q24;q32)	m	8	ACC 160	DSMZ ^c^
9	CA-46	−	inv-dup	t(8;14)(q24;q32)	n.a.	n.a.	ACC 73	DSMZ ^c^
10	CW698	−	inv-dup	t(8;14)(q24;q32)	n.a.	n.a.	−	Prof. G. Klein ^b^
11	BL-30	−	dup	t(8;14)(q24;q32)	m	19	IARC116A	IARC ^d^
12	BL-31	−	dup	t(8;14)(q24;q32)	m	14	IARC142A	IARC ^d^
13	BL-70	−	dup	t(8;14)(q24;q32)	m	16	ACC 233	DSMZ ^c^
14	Loukes	−	dup	t(8;14)(q24;q32)	n.a.	n.a.	−	Prof. G. Klein ^b^
15	MN-60	−	dup	t(8;14)(q24;q32)	m	20	ACC 138	DSMZ ^c^
16	Tanoue	−	dup	t(8;14)(q24;q32)	m	11	ACC 399	DSMZ ^c^
17	BL-49	−	del	t(8;22)(q24;q11)	m	3	−	Prof. G. Klein ^b^
18	AG876	+	−	t(8;14)(q24;q32)	m	8	−	Prof. G. Klein ^b^
19	Akuba	+	−	t(8;22)(q24;q11)	n.a.	n.a.	−	Prof. G. Klein ^b^
20	BL-18	+	−	t(8;14)(q24;q32)	m	3	−	Prof. G. Klein ^b^
21	BL-60	+	−	t(8;22)(q24;q11)	f	4	−	Prof. G. Klein ^b^
22	DAUDI	+	−	t(8;14)(q24;q32)	m	16	ACC 78	DSMZ ^c^
23	DOHH-2	+	−	t(8;14;18)(q24;q32;q21)	f	9	ACC 47	DSMZ ^c^
24	Jijoye M13	+	−	t(8;14)(q24;q32)	m	7	−	Prof. G. Klein ^b^
25	LY-91	+	−	t(2;8)(p12;q24)	f	7	−	Prof. G. Klein ^b^
26	Naliaka	+	−	t(8;14)(q24;q32)	n.a.	n.a.	−	Prof. G. Klein ^b^
27	Rael	+	−	t(8;14)(q24;q32)	n.a.	n.a.	−	Prof. G. Klein ^b^
28	Raji	+	−	t(8;14)(q24;q32)	m	12	ACC 319	DSMZ ^c^
29	Switzer	+	−	t(8;14)(q24;q32)	m	16	−	Prof. G. Klein ^b^
30	BL-16	+	par-tri	t(8;14)(q24;q32)	f	5	−	Prof. G. Klein ^b^
31	JI	+	par-tri	t(2;8)(p12;q24)	f	34	−	Prof. G. Klein ^b^
32	LY-47	+	par-tri	t(8;22)(q24;q11)	m	n.a.	−	Prof. G. Klein ^b^
33	Seraphina	+	par-tri	t(8;14)(q24;q32)	f	7	−	Prof. G. Klein ^b^
34	Silfere	+	par-tri	t(8;14)(q24;q32)	f	6	−	Prof. G. Klein ^b^
35	LY-66	+	inv-dup	t(2;8)(p12;q24)	m	13	−	Prof. G. Klein ^b^
36	NAMALWA	+	inv-dup	t(8;14)(q24;q32)	f	3	ACC 24	DSMZ ^c^
37	KK124	+	dup; del	t(8;22)(q24;q11)	m	n.a.	−	Prof. G. Klein ^b^

All 37 BL-CLs listed here were used for qPCR experiments. ^a^ Dark grey highlighted BL-CLs were used for both qPCR and miRNA analysis. ^b^ Prof. G. Klein (Department of Microbiology & Tumor Biology, Karolinska Institute, Stockholm, Sweden). ^c^ DSMZ-German Collection of Microorganisms and Cell Cultures (DSMZ, Braunschweig, Germany). ^d^ International Agency for Research on Cancer (IARC, Lyon, France). EBV status is indicated as − (negative) and + (positive) in the third column. A minus (−) in the fourth column indicates no 1q gains. Del: Deletion, dup: Duplication, inv-dup: Inverted duplication, Par-tri: Partial trisomy, m: Male. f: Female. n.a.: Not available.

## Data Availability

Not applicable.

## Supplementary Materials

The following supporting information can be downloaded at: https://www.mdpi.com/article/10.3390/v15091808/s1, “Supplemental_data_Akyuz_2023”. Table S1: List of the FISH probes used in this study; Table S2: List of the qPCR primers used in this study; Table S3: Summary of the significantly dysregulated miRNA regarding their EBV status; Table S4: Summary of the significantly dysregulated miRNA regarding their 1q gain status; Table S5: Expression profiles of the significantly dysregulated miRNAs in different cancer entities used in our study. Please note that the references [85,86]-are cited only in the “Supplementary Materials” section and [31,32,70,71,74,80,87,88,89,90,91,92,93,94,95,96,97,98,99,100,101,102,103,104,105,106,107,108,109,110,111,112,113,114,115,116,117,118,119,120,121,122,123,124,125,126,127,128,129,130,131,132,133,134,135,136,137,138,139,140,141,142,143,144,145,146,147,148,149,150,151,152,153,154,155,156,157,158,159,160,161,162,163,164,165,166,167,168,169,170,171,172,173,174,175,176,177,178,179,180,181,182,183,184,185,186,187,188,189,190,191,192,193,194,195,196,197,198,199,200,201,202,203,204,205] only in the supplementary Table S5. The reference [34] is cited both in the main text and in the “Supplementary Materials” section. The references [25,31,32,69,70,71,72,74,80,82] are cited both in the main text and in the supplementary Table S5.
