# Supplementary material for: EBV and 1q Gains Affect Gene and miRNA Expression in Burkitt Lymphoma
Source: Viruses. 2023 Aug 25;15(9):1808. doi: 10.3390/v15091808 (PMC10537407; doi:10.3390/v15091808)
Supplement: Supplementary file 1 [file viruses-15-01808-s001.zip › viruses-2539385-supplementary.pdf]

SUPPLEMENTAL DATA

SUPPLEMENTAL MATERIALS AND METHODS

Cell culture and preparation of the specimens

BL cell lines (BL-CLs) were cultured in RPMI 1640 (Gibco-Invitrogen, Karlsruhe, Germany) supplemented with antibiotics (Gibco-Invitrogen) and 10% fetal bovine serum (FBS) (Gibco-Invitrogen). Cells were treated with Colcemid (0.02 µg/ml) (Gibco-Invitrogen) overnight when they were growing rapidly. BL-CLs and primary material were harvested and fixed with methanol-acetic acid (3:1 v/v) by conventional methods [1].

Multicolor Fluorescence In Situ Hybridization

We performed multicolor fluorescence in situ hybridization (mFISH) using the 24Xcyte color kit for human chromosomes (MetaSystems, Altlussheim, Germany) according to the supplier's recommendations. Briefly, metaphases were hybridized with 24 different chromosome painting probes with specific fluorochrome combinations for all human chromosomes simultaneously. Each paint was labeled with one of the five different fluorochromes (DEAC, FITC, Spectrum Orange, Texas Res, Cy5) or with a unique combination of them. Metaphase chromosomes were counterstained with 4,6-diamino-2-phenylindole (DAPI) (Boehringer/Roche, Mannheim, Germany). After hybridization, gray-scale images of the fluorochromes were acquired using an epifluorescence microscope (Carl Zeiss, Jena, Germany) equipped with a high-resolution cooled CCD camera (Photometrics, München, Germany). A 24-pseudocolor image was built up by overlay of the gray-scale images and analysed by the MetaSystems Isis software package (MetaSystems). Karyotypes were described according to the International System for Human Cytogenetic Nomenclature (ISCN, 2020) [2] and revised using the "CyDAS Online Analysis Site" [3].

Probe selection for metaphase FISH mapping of 1q abnormalities

To determine the exact position of the breakpoints detected by mFISH and possible target genes on 1q, and to uncover cryptic 1q rearrangements, we applied FISH with 37 specific probes covering the whole 1q (supplemental Table S1). Dual-color metaphase FISH was performed on chromosome preparations from all BL patients using two differentially labeled bacterial artificial chromosome (BAC) clones hybridizing to 1q. Biotin-16- or digoxigenin-11-dUTP labeling of DNA by nick translation and FISH was performed according to standard methods as previously described [1, 4]. Briefly a minimum of 10 well-conserved and complete abnormal metaphases were evaluated for each probe. The metaphase chromosomes/nuclei were counterstained with DAPI (Boehringer/Roche). Karyotypes were described according to the ISCN [2]. Cytogenetic position of differentially labeled BAC and P1 artificial chromosomes (PAC) clones on 1q were confirmed by hybridization to normal metaphase spreads obtained from phytohemagglutinin-stimulated peripheral blood lymphocytes of a healthy donor.

For the metaphase FISH analyses, 37 partially overlapping BAC and PAC clones spanning the whole chromosomal band 1q were selected from information archived (on January 2005) by the National Center for Biotechnology Information (NCBI) (http://www.ncbi.nlm.nih.gov/) and obtained from the RPCI-1, -3, -4 and -11 libraries (Roswell Park Cancer Institute, Buffalo, NY). Probes selected for this study were summarized in supplemental Table S1 in order to their cytogenetic positions on 1q.

SUPPLEMENTAL TABLES

Supplemental Table S1: List of the FISH probes used in this study.

Clone	Covered gene region	Mapped to
RP4-666F24	*TSHB*/*TSPAN-2*	1p13.1
pUC1.77	Chromosome 1-specific satellite III	1q12
RP11-326G21	*PDE4DIP*	1q21
RP11-441L11	*BCL9*	1q21.1
RP11-123P3	*BCL9*	1q21.1
RP11-533N14	*BCL9*/*ACP6*	1q21.1
RP11-433J22	*GJA5*	1q21.1
RP11-54A4	*MCL1*	1q21.2
RP11-42A2	Telomeric to *MLLT11* (*AF1q)*	1q21.2
RP11-498A2	Centromeric to *MUC1*	1q22
RP11-98F1	Telomeric to *MUC1*	1q22
RP11-110J1	*ETV3*	1q23.1
RP11-91G5	*FCRL4*	1q23.1
RP11-130F5	*UHMK1*	1q23.3
RP11-506O24	*PBX1*	1q23.3
RP3-395P12	*TNFSF4*	1q24-q25
RP11-152A16	*ABL2*	1q24.1-q24.3
RP11-46A10	*STX6/XPR1*	1q25.2-q31.1
RP11-181K3	*LAMC1/LAMC2/NMNAT2*	1q31
RP1-53A198	*EDEM3*	1q31.1
RP11-339I2	*TPR*	1q31.1
RP11-547I7	*FAM5C*	1q31.1
RP11-198A7	*RGS18*	1q31.2
RP11-552K17	*KCNT2*	1q31.3
RP11-553K8	*ATP6V1G3/PTPRC*	1q31.3
RP11-31E23	Telomeric to *PTPRC*	1q31.3
RP11-343H5	*LGTN/DYRK3/MAPKAPK2*/*IL-10*	1q32.1
RP11-354K1	*NEK2/*telomeric to *TRAF5*	1q32.2
RP11-122M14	*NEK2/LPGAT1*	1q32.2
RP11-264J19	*PPP2R5A*	1q32.2-q32.3
RP11-90A5	*ATF3/FAM71A*	1q32.3
RP11-74E6	*SMYD2* (3' extreme)*/PTPN14*	1q32.3
RP11-66M7	*ESRRG*	1q41
RP11-135J2	*LYPLAL1*	1q41
RP11-192M1	*LEFTY1/PYCR2/LEFTY2*	1q42.1
RP11-91M14	*FMN2*	1q43
RP11-462C5	*OR2M2/OR2M3/OR2M4/OR2M7/OR2T12/OR2T33*	1q44

The probes were listed according to their cytogenetic positions on 1q. The clone name, covered gene region and mapped cytogenetic band are given according to information archived on January 2005 by the NCBI.

Supplemental Table S2: List of the qPCR primers used in this study.

Gene	Forward primer	Reverse primer
HMBS	5'-TGAGAGTGATTCGCGTGGGTAC-3'	5'-CCCTGTGGTGGACATAGCAATG-3'
MLLT11 (AF1q)	QuantiTect Primer Assay Hs_MLLT11_1_SG	
ARNT	QuantiTect Primer Assay Hs_ARNT_1_SG	
BCL9	QuantiTect Primer Assay Hs_BCL9_2_SG	
FCRL5 (IRTA2)	QuantiTect Primer Assay Hs_FCRL5_1_SG	
MCL1	QuantiTect Primer Assay Hs_MCL1_1_SG	
PBXIP1	QuantiTect Primer Assay Hs_PBXIP1_1_SG	

Primer for *HMBS* were obtained from Eurofins (Ebersberg, Germany) and QuantiTect Primer Assay from QIAGEN (Hilden, Germany).

Supplemental Table S3: Summary of the significantly dysregulated miRNA regarding their EBV status.

	In EBV+ BL-CLs	In EBV- BL -CLs
**Upregulated in BL-CLs with 1q gains and downregulated in BL-CLs without 1q gains**	hsa-miR-17	**hsa-miR-9**
	hsa-miR-17*	**hsa-miR-9***
	hsa-miR-18a	hsa-miR-96
	hsa-miR-18b	hsa-miR-148a
	hsa-miR-19b	**hsa-miR-181a**
	hsa-miR-20a	**hsa-miR-181b**
	hsa-miR-20a*	hsa-miR-182
	hsa-miR-20b	hsa-miR-183
	hsa-miR-23a	hsa-miR-198
	hsa-miR-27a	hsa-miR-335
	hsa-miR-33a	hsa-miRPlus-B1114
	hsa-miR-92a	hsa-miRPlus-E1117
	**hsa-miR-92b**	hsa-miRPlus-E1168
	hsa-miR-93	
	hsa-miR-99a	
	hsa-miR-100	
	hsa-miR-106a	
	hsa-miR-125b	
	**hsa-miR-181a**	
	**hsa-miR-181b**	
	hsa-miR-183	
	hsa-miR-193a-3p	
	hsa-miRPlus-E1038	
	hsa-miRPlus-F1181	
**Downregulated in BL-CLs with 1q gains and upregulated in BL-CLs without 1q gains**	**hsa-miR-9***	hsa-miR-22
	hsa-miR-124*	hsa-miR-28-5p
	hsa-miR-492	hsa-miR-193b
	hsa-miR-542-5p	
	hsa-miR-583	
	hsa-miR-675	
	hsa-miR-1275	
	hsa-miRPlus-B1114	
	hsa-miRPlus-F1216	
**Stronger downregulated in BL-CLs with 1q gains**	hsa-miR-138	hsa-miR-155
	hsa-miR-1290	
**Stronger downregulated in BL-CLs without 1q gains**	hsa-miR-96	hsa-miR-1290
	hsa-miR-182	
	hsa-miR-339-5p	

Five 1q resident miRNAs are highlighted in bold letters. Three miRNAs dysregulated depending on 1q gains but independent of EBV are underlined. BL-CLs: Burkitt lymphoma cell lines. EBV-: EBV-negative BL-CLs. EBV+: EBV-positive BL-CLs. miRNA*: Passenger miRNA strand.

Supplemental Table S4: Summary of the significantly dysregulated miRNA regarding their 1q gain status.

	In BL-CLs with 1q gains	In BL-CLs without 1q gains
**Upregulated in EBV+ and downregulated in EBV- BL-CLs**	hsa-miR-7	***hsa-miR-21***
	hsa-miR-21	hsa-miR-105
	hsa-miR-22	***hsa-miR-155***
	hsa-miR-24	***hsa-miR-155****
	hsa-miR-27a	hsa-miR-198
	hsa-miR-99a	***hsa-miR-221***
	hsa-miR-100	***hsa-miR-222***
	hsa-miR-125b	hsa-miR-542-5p
	hsa-miR-155	hsa-miR-583
	hsa-miR-155*	hsa-miR-675
	hsa-miR-221	hsa-miR-1275
	hsa-miR-222	hsa-miRPlus-B1114
	hsa-miR-487b	hsa-miRPlus-E1168
	hsa-miR-519d	hsa-miRPlus-F1193
	hsa-miR-1265	
	hsa-miRPlus-E1013	
	hsa-miRPlus-E1038	
	hsa-miRPlus-E1103	
**Downregulated in EBV+ and upregulated in EBV- BL-CLs**	**hsa-miR-9**	hsa-miR-15a
	**hsa-miR-9***	hsa-miR-28-5p
	hsa-miR-138	hsa-miR-33a
	hsa-miR-335	hsa-miR-339-5p
	hsa-miR-1246	
	hsa-miRPlus-B1114	
**Stronger downregulated in EBV+ BL-CLs**	hsa-miR-675	hsa-miR-96
	hsa-miR-1290	hsa-miR-125b
		hsa-miR-182
**Stronger downregulated in EBV- BL-CLs**		hsa-miRPlus-A1027

1q resident miRNAs are highlighted in bold letters. Five miRNAs dysregulated depending on EBV but independent of 1q gains are highlighted in italic bold letters. BL-CLs: Burkitt lymphoma cell lines. EBV-: EBV-negative BL-CLs. EBV+: EBV-positive BL-CLs. miRNA*: Passenger miRNA strand.

Supplemental Table S5: Expression profiles of the in our study significantly dysregulated miRNAs in different cancer entities.

**No.**	**miRNA**	**Expression in different cancer entities according to the supplemental references[a]**	**Supplemental references[b]**
**1**	hsa-miRPlus-B1114	gguccacaggggagauagg	NRY
**2**	hsa-miR-9*	-up- and downregulated in AML subtypes and different cancer entities<br>-upregulated in FL<br>-downregulated in **BL**, ALL	**[5-8]**
**3**	hsa-miR-1290	-upregulated in AML, ALL, and different cancer types like colorectal cancer, pancreatic cancer, lung cancer	**[9, 10]**
**4**	hsa-miR-96	-upregulated in hepatocellular carcinoma, malignant breast cancer and **BL**<br>-downregulated in AML and CML	**[11-16]**
**5**	hsa-miR-182	-upregulated in AML and **BL**<br>-downregulated in ALL and osteosarcoma,	**[15-20]**
**6**	hsa-miR-181a	-upregulated in AML, MM and ALL<br>-downregulated in prostate cancer	**[21-24]**
**7**	hsa-miR-181b	-upregulated in MM<br>-downregulated in **BL**, B-ALL and CLL	**[23, 25-27]**
**8**	hsa-miR-183	-upregulated in **BL**, MM, AML and classical Hodgkin lymphoma	**[15, 16, 20, 23, 28]**
**9**	hsa-miR-125b	-upregulated in MM, T-ALL and AML<br>-downregulated in CLL and oral carcinoma	**[23, 29-33]**
**10**	hsa-miR-675	-downregulated in AML	**[34]**
**11**	hsa-miR-27a	-upregulated in gastric cancer and MM<br>-downregulated in AML and ALL	**[23, 35-37]**
**12**	hsa-miR-99a	-upregulated in T-ALL, leukemia stem cells of AML and AML<br>-downregulated in prostate cancer stem cells and ALL	**[29, 30, 38-40]**
**13**	hsa-miR-100	-upregulated MM, AML and SMZL<br>-downregulated in prostate cancer stem cells and ALL	**[23, 30, 38, 40, 41]**
**14**	hsa-miRPlus-E1038	gcaugagugguucaguggu	NRY
**15**	hsa-miR-138	-downregulated in CLL and HCV-positive DLBCL	**[42, 43]**
**16**	hsa-miR-33a	-downregulated in lung cancer and triple-negative breast cancer	**[44, 45]**

**17**	hsa-miR-339-5p	-downregulated in AML	**[46]**
**18**	hsa-miR-542-5p	-upregulated in T-ALL	**[47]**
**19**	hsa-miR-583	-downregulated in recurrent prostate cancer	**[48]**
**20**	hsa-miR-1275	-upregulated in CML<br>-downregulated in AML and **BL**	**[20, 49, 50]**
**21**	hsa-miR-17	-upregulated in hematologic and solid cancer<br>-downregulated in CLL and oral carcinoma	**[23, 32, 33, 51-55]**
**22**	hsa-miR-17*	-upregulated in hematologic and solid cancer<br>-downregulated in prostate cancer and FL	**[7, 33, 52, 56]**
**23**	hsa-miR-18a	-upregulated in hematologic and solid cancer	**[33, 52, 53, 57]**
**24**	hsa-miR-20a	-upregulated in hematologic and solid cancer<br>-downregulated in oral carcinoma	**[7, 8, 23, 32, 33, 52-55]**
**25**	hsa-miR-20a*	-upregulated in hematologic and solid cancer<br>-downregulated in gastric cancer	**[33, 52, 58]**
**26**	hsa-miR-19b	-upregulated in hematologic and solid cancer	**[20, 23, 33, 52, 53, 55]**
**27**	hsa-miR-92a	-upregulated in hematologic and solid cancer	**[20, 23, 33, 52, 53, 55]**
**28**	hsa-miR-106a	-upregulated in **BL**, hepatocellular carcinoma, breast cancer and MCL	**[53, 59, 60]**
**29**	hsa-miR-18b	-upregulated in **BL**, MCL and breast cancer	**[53, 61]**
**30**	hsa-miR-20b	-upregulated in **BL**, CLL and FL	**[7, 53, 62]**
**31**	hsa-miR-93	-upregulated in MCL and breast cancer	**[60]**
**32**	hsa-miR-23a	-upregulated in AML and MM<br>-downregulated in CML and **BL**	**[23, 53, 63, 64]**
**33**	hsa-miR-92b	-upregulated in **BL**, GBC and prostate cancer<br>-downregulated in MCL	**[20, 65-67]**
**34**	hsa-miR-193a-3p	-upregulated in renal cell carcinoma and MM<br>-downregulated in NSCLC and AML	**[23, 68-70]**
**35**	hsa-miRPlus-F1181	ugaaaugcaaauccaugcaa	NRY
**36**	hsa-miR-124*	-upregulated in AML<br>-downregulated in glioma	**[54, 71]**
**37**	hsa-miR-492	-upregulated in hepatoblastoma and bladder cancer	**[72-74]**

		-downregulated in Colorectal cancer	
**38**	hsa-miRPlus-F1216	ggagagggaaaagaaaag	NRY
**39**	hsa-miR-155	-upregulated in gastric cancer, SMZL, oral carcinoma and some B-cell lymphomas (CLL, AML, **BL**)<br>-downregulated in CML and ALL and **BL**	**[20, 32, 33, 35, 41, 52, 53, 75-83]**
**40**	hsa-miR-9	-up- and downregulated in different AML subtypes and cancer entities<br>-upregulated in FL<br>-downregulated in MM, ALL	**[5-7]**
**41**	hsa-miR-335	-upregulated in AML and multiple myeloma<br>-downregulated in MM	**[23, 84, 85]**
**42**	hsa-miR-22	-upregulated in CLL and **BL**<br>-downregulated in T-ALL and AML	**[20, 33, 86-88]**
**43**	hsa-miR-198	-upregulated in retinoblastoma<br>-downregulated in pancreatic cancer, NSCLC and MM	**[23, 89-91]**
**44**	hsa-miRPlus-E1168	cggcgggagcccgggg	NRY
**45**	hsa-miR-28-5p	-upregulated in glioblastoma<br>-downregulated in gastric cancer, **BL**, DLBCL, follicular lymphoma, and CLL	**[20, 92-95]**
**46**	hsa-miR-148a	-upregulated in **BL**, ALL and AML<br>-downregulated in breast cancer	**[20, 78, 96, 97]**
**47**	hsa-miRPlus-E1117	aagacgagaagacccuauggagcuu	NRY
**48**	hsa-miR-193b	-upregulated in SMZL<br>-downregulated in AML, melanoma	**[41, 98, 99]**
**49**	hsa-miR-7	-upregulated in breast cancer and renal cell carcinoma<br>-downregulated in T-ALL and gastric cancer stem cells	**[100-103]**
**50**	hsa-miR-24	-upregulated in acute leukemia and pancreatic cancer<br>-downregulated in gastric cancer	**[78, 104-106]**
**51**	hsa-miR-487b	-upregulated in osteosarcoma<br>-downregulated in colorectal cancer and neuroblastoma	**[107-109]**
**52**	hsa-miR-519d	-upregulated in melanoma<br>-downregulated in breast cancer stem cells	**[110, 111]**
**53**	hsa-miR-1265	-downregulated in gastric cancer	**[112]**

**54**	hsa-miRPlus-E1013	ucccuucguggucgcc	NRY
**55**	hsa-miRPlus-E1103	ucccugguggucuagu	NRY
**56**	hsa-miR-1246	-upregulated in T-ALL oral carcinoma and pancreatic cancer	**[32, 113, 114]**
**57**	hsa-miR-21	-upregulated in **BL**, MM, ALL, SMZL, oral carcinoma, CLL and many other cancer entities<br>-downregulated in **BL**	**[20, 23, 32, 33, 41, 52, 53, 78, 80, 81, 115]**
**58**	hsa-miR-155*	-upregulated in glioma, breast cancer and hepatocellular carcinoma<br>-downregulated in mantle cell lymphoma and other non-Hodgkin's lymphomas	**[116-119]**
**59**	hsa-miR-221	-upregulated in T-ALL, AML, CLL, cervical cancer, MM and many other cancer entities<br>-downregulated in erythroblastic leukemia and **BL**	**[20, 23, 52, 120-125]**
**60**	hsa-miR-222	-upregulated in CLL and many other cancer entities<br>-downregulated in erythroblastic leukemia and **BL**	**[20, 52, 53, 123-125]**
**61**	hsa-miR-105	-upregulated in esophageal cancer and many different cancer entities<br>-downregulated in gastric cancer and many different cancer entities	**[52, 126-128]**
**62**	hsa-miRPlus-F1193	cugggugagagcgggagg	NRY
**63**	hsa-miR-15a	-downregulated in **BL**, MCL, CLL, AML and many different cancer entities	**[33, 52, 79, 129-132]**
**64**	hsa-miRPlus-A1027	auguugggagcgggcagguugg	NRY

All miRNAs are numbered according to their appearance in Figure 3 (main manuscript). [a]: A report in Burkitt lymphoma is highlighted in bold letters (**BL**) and since miRPlus are not published elsewhere, the appropriate miRNA sequence is indicated here. [b]: Please note that all references are numbered according to their appearance in this table and are summarized in supplemental reference list. NRY: Not reported yet. (T-)ALL: (T cell-). Acute lymphocytic leukemia. AML: Acute myeloid leukemia. CLL: Chronic lymphocytic leukemia. CML: Chronic myeloid leukemia. GBC: Gallbladder cancer. MCL: Mantel cell lymphoma. MM: Multiple myeloma. NSCLC: Non-small cell lung cancer. SMZL: Splenic marginal zone lymphoma.

SUPPLEMENTAL REFERENCES

1. Dierlamm, J.; Baens, M.; Stefanova-Ouzounova, M.; Hinz, K.; Wlodarska, I.; Maes, B.; Steyls, A.; Driessen, A.; Verhoef, G.; Gaulard, P.; Hagemeijer, A.; Hossfeld, D. K.; De Wolf-Peeters, C.; Marynen, P., Detection of t(11;18)(q21;q21) by interphase fluorescence in situ hybridization using API2 and MLT specific probes. *Blood* **2000,** 96, (6), 2215-8.
2. McGowan-Jordan J, H. R., Moore S, editors., *ISCN 2020 An International System for Human Cytogenomic Nomenclature (2020)*. Karger: Basel, 2020.
3. Hiller, B.; Bradtke, J.; Balz, H.; Rieder, H. CyDAS Online Analysis Site.
4. Dierlamm, J.; Wlodarska, I.; Michaux, L.; La Starza, R.; Zeller, W.; Mecucci, C.; Van den Berghe, H., Successful use of the same slide for consecutive fluorescence in situ hybridization experiments. *Genes, chromosomes & cancer* **1996,** 16, (4), 261-4.
5. Khosravi, A.; Alizadeh, S.; Jalili, A.; Shirzad, R.; Saki, N., The impact of Mir-9 regulation in normal and malignant hematopoiesis. *Oncology reviews* **2018,** 12, (1), 348.
6. Nowek, K.; Wiemer, E. A. C.; Jongen-Lavrencic, M., The versatile nature of miR-9/9(*) in human cancer. *Oncotarget* **2018,** 9, (29), 20838-20854.
7. Wang, W.; Corrigan-Cummins, M.; Hudson, J.; Maric, I.; Simakova, O.; Neelapu, S. S.; Kwak, L. W.; Janik, J. E.; Gause, B.; Jaffe, E. S.; Calvo, K. R., MicroRNA profiling of follicular lymphoma identifies microRNAs related to cell proliferation and tumor response. *Haematologica* **2012,** 97, (4), 586-94.
8. Onnis, A.; De Falco, G.; Antonicelli, G.; Onorati, M.; Bellan, C.; Sherman, O.; Sayed, S.; Leoncini, L., Alteration of microRNAs regulated by c-Myc in Burkitt lymphoma. *PloS one* **2010,** 5, (9).
9. Sun, Y.; Ju, X. L.; Li, D.; Zhou, P. P.; Li, X.; Luo, R. H., miR-1290 promotes proliferation and suppresses apoptosis in acute myeloid leukemia by targeting FOXG1/SOCS3. *Journal of biological regulators and homeostatic agents* **2019,** 33, (6), 1703-1713.
10. Zhou, W.; Wang, S.; Ying, Y.; Zhou, R.; Mao, P., miR-196b/miR-1290 participate in the antitumor effect of resveratrol via regulation of IGFBP3 expression in acute lymphoblastic leukemia. *Oncology reports* **2017,** 37, (2), 1075-1083.
11. Hu, N.; Chen, L.; Wang, C.; Zhao, H., MALAT1 knockdown inhibits proliferation and enhances cytarabine chemosensitivity by upregulating miR-96 in acute myeloid leukemia cells. *Biomedicine & pharmacotherapy = Biomedecine & pharmacotherapie* **2019,** 112, 108720.
12. Huang, T.; Fu, Y.; Wang, S.; Xu, M.; Yin, X.; Zhou, M.; Wang, X.; Chen, C., miR-96 acts as a tumor suppressor via targeting the BCR-ABL1 oncogene in chronic myeloid leukemia blastic transformation. *Biomedicine & pharmacotherapy = Biomedecine & pharmacotherapie* **2019,** 119, 109413.
13. Iwai, N.; Yasui, K.; Tomie, A.; Gen, Y.; Terasaki, K.; Kitaichi, T.; Soda, T.; Yamada, N.; Dohi, O.; Seko, Y.; Umemura, A.; Nishikawa, T.; Yamaguchi, K.; Moriguchi, M.; Konishi, H.; Naito, Y.; Itoh, Y., Oncogenic miR-96-5p inhibits apoptosis by targeting the caspase-9 gene in hepatocellular carcinoma. *International journal of oncology* **2018,** 53, (1), 237-245.
14. Xie, W.; Sun, F.; Chen, L.; Cao, X., miR-96 promotes breast cancer metastasis by suppressing MTSS1. *Oncology letters* **2018,** 15, (3), 3464-3471.
15. Xie, L.; Ushmorov, A.; Leithäuser, F.; Guan, H.; Steidl, C.; Färbinger, J.; Pelzer, C.; Vogel, M. J.; Maier, H. J.; Gascoyne, R. D.; Möller, P.; Wirth, T., FOXO1 is a tumor suppressor in classical Hodgkin lymphoma. *Blood* **2012,** 119, (15), 3503-11.

16. Oussaief, L.; Fendri, A.; Chane-Woon-Ming, B.; Poirey, R.; Delecluse, H. J.; Joab, I.; Pfeffer, S., Modulation of MicroRNA Cluster miR-183-96-182 Expression by Epstein-Barr Virus Latent Membrane Protein 1. *Journal of virology* **2015,** 89, (23), 12178-88.
17. Hu, J.; Lv, G.; Zhou, S.; Zhou, Y.; Nie, B.; Duan, H.; Zhang, Y.; Yuan, X., The Downregulation of MiR-182 Is Associated with the Growth and Invasion of Osteosarcoma Cells through the Regulation of TIAM1 Expression. *PloS one* **2015,** 10, (5), e0121175.
18. Zhang, S.; Zhang, Q.; Shi, G.; Yin, J., MiR-182-5p regulates BCL2L12 and BCL2 expression in acute myeloid leukemia as a potential therapeutic target. *Biomedicine & pharmacotherapy = Biomedecine & pharmacotherapie* **2018,** 97, 1189-1194.
19. Piatopoulou, D.; Avgeris, M.; Marmarinos, A.; Xagorari, M.; Baka, M.; Doganis, D.; Kossiva, L.; Scorilas, A.; Gourgiotis, D., miR-125b predicts childhood acute lymphoblastic leukaemia poor response to BFM chemotherapy treatment. *British journal of cancer* **2017,** 117, (6), 801-812.
20. Oduor, C. I.; Kaymaz, Y.; Chelimo, K.; Otieno, J. A.; Ong'echa, J. M.; Moormann, A. M.; Bailey, J. A., Integrative microRNA and mRNA deep-sequencing expression profiling in endemic Burkitt lymphoma. *BMC cancer* **2017,** 17, (1), 761.
21. Liu, X.; Liao, W.; Peng, H.; Luo, X.; Luo, Z.; Jiang, H.; Xu, L., miR-181a promotes G1/S transition and cell proliferation in pediatric acute myeloid leukemia by targeting ATM. *Journal of cancer research and clinical oncology* **2016,** 142, (1), 77-87.
22. Verduci, L.; Azzalin, G.; Gioiosa, S.; Carissimi, C.; Laudadio, I.; Fulci, V.; Macino, G., microRNA-181a enhances cell proliferation in acute lymphoblastic leukemia by targeting EGR1. *Leukemia research* **2015,** 39, (4), 479-85.
23. Pichiorri, F.; Suh, S. S.; Ladetto, M.; Kuehl, M.; Palumbo, T.; Drandi, D.; Taccioli, C.; Zanesi, N.; Alder, H.; Hagan, J. P.; Munker, R.; Volinia, S.; Boccadoro, M.; Garzon, R.; Palumbo, A.; Aqeilan, R. I.; Croce, C. M., MicroRNAs regulate critical genes associated with multiple myeloma pathogenesis. *Proceedings of the National Academy of Sciences of the United States of America* **2008,** 105, (35), 12885-90.
24. Shen, H.; Weng, X. D.; Liu, X. H.; Yang, D.; Wang, L.; Guo, J.; Wang, M.; Wang, X.; Diao, C. H., miR-181a-5p is downregulated and inhibits proliferation and the cell cycle in prostate cancer. *International journal of clinical and experimental pathology* **2018,** 11, (8), 3969-3976.
25. Zhou, G.; Cao, Y.; Dong, W.; Lin, Y.; Wang, Q.; Wu, W.; Hua, X.; Ling, Y.; Xie, X.; Hu, S.; Cen, J.; Gu, W., The clinical characteristics and prognostic significance of AID, miR-181b, and miR-155 expression in adult patients with de novo B-cell acute lymphoblastic leukemia. *Leukemia & lymphoma* **2017,** 58, (9), 1-9.
26. Visone, R.; Veronese, A.; Rassenti, L. Z.; Balatti, V.; Pearl, D. K.; Acunzo, M.; Volinia, S.; Taccioli, C.; Kipps, T. J.; Croce, C. M., miR-181b is a biomarker of disease progression in chronic lymphocytic leukemia. *Blood* **2011,** 118, (11), 3072-9.
27. Li, J. G.; Ding, Y.; Huang, Y. M.; Chen, W. L.; Pan, L. L.; Li, Y.; Chen, X. L.; Chen, Y.; Wang, S. Y.; Wu, X. N., FAMLF is a target of miR-181b in Burkitt lymphoma. *Brazilian journal of medical and biological research = Revista brasileira de pesquisas medicas e biologicas* **2017,** 50, (6), e5661.
28. Wang, X.; Zuo, D.; Yuan, Y.; Yang, X.; Hong, Z.; Zhang, R., MicroRNA-183 promotes cell proliferation via regulating programmed cell death 6 in pediatric acute myeloid leukemia. *Journal of cancer research and clinical oncology* **2017,** 143, (1), 169-180.
29. Renou, L.; Boelle, P. Y.; Deswarte, C.; Spicuglia, S.; Benyoucef, A.; Calvo, J.; Uzan, B.; Belhocine, M.; Cieslak, A.; Landman-Parker, J.; Baruchel, A.; Asnafi, V.; Pflumio, F.; Ballerini, P.; Naguibneva, I., Homeobox protein TLX3 activates miR-125b expression to promote T-cell acute lymphoblastic leukemia. *Blood advances* **2017,** 1, (12), 733-747.

30. Zhang, H.; Luo, X. Q.; Zhang, P.; Huang, L. B.; Zheng, Y. S.; Wu, J.; Zhou, H.; Qu, L. H.; Xu, L.; Chen, Y. Q., MicroRNA patterns associated with clinical prognostic parameters and CNS relapse prediction in pediatric acute leukemia. *PloS one* **2009,** 4, (11), e7826.
31. Tili, E.; Michaille, J. J.; Luo, Z.; Volinia, S.; Rassenti, L. Z.; Kipps, T. J.; Croce, C. M., The down-regulation of miR-125b in chronic lymphocytic leukemias leads to metabolic adaptation of cells to a transformed state. *Blood* **2012,** 120, (13), 2631-8.
32. Dioguardi, M.; Caloro, G. A.; Laino, L.; Alovisi, M.; Sovereto, D.; Crincoli, V.; Aiuto, R.; Coccia, E.; Troiano, G.; Lo Muzio, L., Circulating miR-21 as a Potential Biomarker for the Diagnosis of Oral Cancer: A Systematic Review with Meta-Analysis. *Cancers* **2020,** 12, (4).
33. Weiss, C. N.; Ito, K., A Macro View of MicroRNAs: The Discovery of MicroRNAs and Their Role in Hematopoiesis and Hematologic Disease. *International review of cell and molecular biology* **2017,** 334, 99-175.
34. Zhang, T. J.; Zhou, J. D.; Zhang, W.; Lin, J.; Ma, J. C.; Wen, X. M.; Yuan, Q.; Li, X. X.; Xu, Z. J.; Qian, J., H19 overexpression promotes leukemogenesis and predicts unfavorable prognosis in acute myeloid leukemia. *Clinical epigenetics* **2018,** 10, 47.
35. Li, Y.; Tian, Z.; Tan, Y.; Lian, G.; Chen, S.; Chen, S.; Li, J.; Li, X.; Huang, K.; Chen, Y., Bmi-1-induced miR-27a and miR-155 promote tumor metastasis and chemoresistance by targeting RKIP in gastric cancer. *Molecular cancer* **2020,** 19, (1), 109.
36. Sun, Y. P.; Lu, F.; Han, X. Y.; Ji, M.; Zhou, Y.; Zhang, A. M.; Wang, H. C.; Ma, D. X.; Ji, C. Y., MiR-424 and miR-27a increase TRAIL sensitivity of acute myeloid leukemia by targeting PLAG1. *Oncotarget* **2016,** 7, (18), 25276-90.
37. Scheibner, K. A.; Teaboldt, B.; Hauer, M. C.; Chen, X.; Cherukuri, S.; Guo, Y.; Kelley, S. M.; Liu, Z.; Baer, M. R.; Heimfeld, S.; Civin, C. I., MiR-27a functions as a tumor suppressor in acute leukemia by regulating 14-3-3θ. *PloS one* **2012,** 7, (12), e50895.
38. Rane, J. K.; Erb, H. H.; Nappo, G.; Mann, V. M.; Simms, M. S.; Collins, A. T.; Visakorpi, T.; Maitland, N. J., Inhibition of the glucocorticoid receptor results in an enhanced miR-99a/100-mediated radiation response in stem-like cells from human prostate cancers. *Oncotarget* **2016,** 7, (32), 51965-51980.
39. Si, X.; Zhang, X.; Hao, X.; Li, Y.; Chen, Z.; Ding, Y.; Shi, H.; Bai, J.; Gao, Y.; Cheng, T.; Yang, F. C.; Zhou, Y., Upregulation of miR-99a is associated with poor prognosis of acute myeloid leukemia and promotes myeloid leukemia cell expansion. *Oncotarget* **2016,** 7, (47), 78095-78109.
40. Li, X. J.; Luo, X. Q.; Han, B. W.; Duan, F. T.; Wei, P. P.; Chen, Y. Q., MicroRNA-100/99a, deregulated in acute lymphoblastic leukaemia, suppress proliferation and promote apoptosis by regulating the FKBP51 and IGF1R/mTOR signalling pathways. *British journal of cancer* **2013,** 109, (8), 2189-98.
41. Arribas, A. J.; Gómez-Abad, C.; Sánchez-Beato, M.; Martinez, N.; Dilisio, L.; Casado, F.; Cruz, M. A.; Algara, P.; Piris, M. A.; Mollejo, M., Splenic marginal zone lymphoma: comprehensive analysis of gene expression and miRNA profiling. *Modern pathology : an official journal of the United States and Canadian Academy of Pathology, Inc* **2013,** 26, (7), 889-901.
42. Berg, V.; Rusch, M.; Vartak, N.; Jüngst, C.; Schauss, A.; Waldmann, H.; Hedberg, C.; Pallasch, C. P.; Bastiaens, P. I.; Hallek, M.; Wendtner, C. M.; Frenzel, L. P., miRs-138 and -424 control palmitoylation-dependent CD95-mediated cell death by targeting acyl protein thioesterases 1 and 2 in CLL. *Blood* **2015,** 125, (19), 2948-57.
43. Augello, C.; Gianelli, U.; Savi, F.; Moro, A.; Bonoldi, E.; Gambacorta, M.; Vaira, V.; Baldini, L.; Bosari, S., MicroRNA as potential biomarker in HCV-associated diffuse large B-cell lymphoma. *Journal of clinical pathology* **2014,** 67, (8), 697-701.

44. Amaar, Y. G.; Reeves, M. E., RASSF1C regulates miR-33a and EMT marker gene expression in lung cancer cells. *Oncotarget* **2019,** 10, (2), 123-132.
45. Guan, X.; Gu, S.; Yuan, M.; Zheng, X.; Wu, J., MicroRNA-33a-5p overexpression sensitizes triple-negative breast cancer to doxorubicin by inhibiting eIF5A2 and epithelial-mesenchymal transition. *Oncology letters* **2019,** 18, (6), 5986-5994.
46. Sun, X.; Liu, H.; Li, T.; Qin, L., MicroRNA-339-5p inhibits cell proliferation of acute myeloid leukaemia by directly targeting SOX4. *Molecular medicine reports* **2018,** 18, (6), 5261-5269.
47. Almeida, R. S.; Costa, E. S. M.; Coutinho, L. L.; Garcia Gomes, R.; Pedrosa, F.; Massaro, J. D.; Donadi, E. A.; Lucena-Silva, N., MicroRNA expression profiles discriminate childhood T- from B-acute lymphoblastic leukemia. *Hematological oncology* **2019,** 37, (1), 103-112.
48. Pashaei, E.; Pashaei, E.; Ahmady, M.; Ozen, M.; Aydin, N., Meta-analysis of miRNA expression profiles for prostate cancer recurrence following radical prostatectomy. *PloS one* **2017,** 12, (6), e0179543.
49. Yang, J. R.; Shi, M. X.; Zeng, Y., LncRNA HAND2-AS1 inhibits proliferation and promotes apoptosis of chronic myeloid leukemia cells by sponging with micRNA-1275. *European review for medical and pharmacological sciences* **2019,** 23, (5), 2103-2111.
50. Ozdogan, H.; Gur Dedeoglu, B.; Oztemur Islakoglu, Y.; Aydos, A.; Kose, S.; Atalay, A.; Yegin, Z. A.; Avcu, F.; Uckan Cetinkaya, D.; Ilhan, O., DICER1 gene and miRNA dysregulation in mesenchymal stem cells of patients with myelodysplastic syndrome and acute myeloblastic leukemia. *Leukemia research* **2017,** 63, 62-71.
51. Mraz, M.; Malinova, K.; Kotaskova, J.; Pavlova, S.; Tichy, B.; Malcikova, J.; Stano Kozubik, K.; Smardova, J.; Brychtova, Y.; Doubek, M.; Trbusek, M.; Mayer, J.; Pospisilova, S., miR-34a, miR-29c and miR-17-5p are downregulated in CLL patients with TP53 abnormalities. *Leukemia* **2009,** 23, (6), 1159-63.
52. Croce, C. M., Causes and consequences of microRNA dysregulation in cancer. *Nature reviews. Genetics* **2009,** 10, (10), 704-14.
53. Iqbal, J.; Shen, Y.; Huang, X.; Liu, Y.; Wake, L.; Liu, C.; Deffenbacher, K.; Lachel, C. M.; Wang, C.; Rohr, J.; Guo, S.; Smith, L. M.; Wright, G.; Bhagavathi, S.; Dybkaer, K.; Fu, K.; Greiner, T. C.; Vose, J. M.; Jaffe, E.; Rimsza, L.; Rosenwald, A.; Ott, G.; Delabie, J.; Campo, E.; Braziel, R. M.; Cook, J. R.; Tubbs, R. R.; Armitage, J. O.; Weisenburger, D. D.; Staudt, L. M.; Gascoyne, R. D.; McKeithan, T. W.; Chan, W. C., Global microRNA expression profiling uncovers molecular markers for classification and prognosis in aggressive B-cell lymphoma. *Blood* **2015,** 125, (7), 1137-45.
54. Li, L.; Zhu, L.; Wang, Y.; Zhou, D.; Zhu, J.; Xie, W.; Ye, X., Profiling of microRNAs in AML cells following overexpression or silencing of the VEGF gene. *Oncology letters* **2017,** 13, (1), 105-110.
55. Robaina, M. C.; Faccion, R. S.; Mazzoccoli, L.; Rezende, L. M.; Queiroga, E.; Bacchi, C. E.; Thomas-Tikhonenko, A.; Klumb, C. E., miR-17-92 cluster components analysis in Burkitt lymphoma: overexpression of miR-17 is associated with poor prognosis. *Annals of hematology* **2016,** 95, (6), 881-91.
56. Xu, Y.; Fang, F.; Zhang, J.; Josson, S.; St Clair, W. H.; St Clair, D. K., miR-17* suppresses tumorigenicity of prostate cancer by inhibiting mitochondrial antioxidant enzymes. *PloS one* **2010,** 5, (12), e14356.
57. Cao, P.; Zhang, M.; Wang, L.; Sai, B.; Tang, J.; Luo, Z.; Shuai, C.; Zhang, L.; Li, Z.; Wang, Y.; Li, G.; Xiang, J., miR-18a reactivates the Epstein-Barr virus through defective DNA damage response and promotes genomic instability in EBV-associated lymphomas. *BMC cancer* **2018,** 18, (1), 1293.
58. Dong, L.; Deng, J.; Sun, Z. M.; Pan, A. P.; Xiang, X. J.; Zhang, L.; Yu, F.; Chen, J.; Sun, Z.; Feng, M.; Xiong, J. P., Interference with the β-catenin gene in gastric cancer

induces changes to the miRNA expression profile. *Tumour biology : the journal of the International Society for Oncodevelopmental Biology and Medicine* **2015,** 36, (9), 6973-83.

59. Luo, L. H.; Jin, M.; Wang, L. Q.; Xu, G. J.; Lin, Z. Y.; Yu, D. D.; Yang, S. L.; Ran, R. Z.; Wu, G.; Zhang, T., Long noncoding RNA TCL6 binds to miR-106a-5p to regulate hepatocellular carcinoma cells through PI3K/AKT signaling pathway. *Journal of cellular physiology* **2020,** 235, (9), 6154-6166.
60. Li, N.; Miao, Y.; Shan, Y.; Liu, B.; Li, Y.; Zhao, L.; Jia, L., MiR-106b and miR-93 regulate cell progression by suppression of PTEN via PI3K/Akt pathway in breast cancer. *Cell death & disease* **2017,** 8, (5), e2796.
61. Fonseca-Sanchéz, M. A.; Pérez-Plasencia, C.; Fernández-Retana, J.; Arechaga-Ocampo, E.; Marchat, L. A.; Rodríguez-Cuevas, S.; Bautista-Piña, V.; Arellano-Anaya, Z. E.; Flores-Pérez, A.; Diaz-Chávez, J.; López-Camarillo, C., microRNA-18b is upregulated in breast cancer and modulates genes involved in cell migration. *Oncology reports* **2013,** 30, (5), 2399-410.
62. Papageorgiou, S. G.; Kontos, C. K.; Tsiakanikas, P.; Stavroulaki, G.; Bouchla, A.; Vasilatou, D.; Bazani, E.; Lazarakou, A.; Scorilas, A.; Pappa, V., Elevated miR-20b-5p expression in peripheral blood mononuclear cells: A novel, independent molecular biomarker of favorable prognosis in chronic lymphocytic leukemia. *Leukemia research* **2018,** 70, 1-7.
63. Hatzl, S.; Geiger, O.; Kuepper, M. K.; Caraffini, V.; Seime, T.; Furlan, T.; Nussbaumer, E.; Wieser, R.; Pichler, M.; Scheideler, M.; Nowek, K.; Jongen-Lavrencic, M.; Quehenberger, F.; Wölfler, A.; Troppmair, J.; Sill, H.; Zebisch, A., Increased Expression of miR-23a Mediates a Loss of Expression in the RAF Kinase Inhibitor Protein RKIP. *Cancer research* **2016,** 76, (12), 3644-54.
64. Xishan, Z.; Xianjun, L.; Ziying, L.; Guangxin, C.; Gang, L., The malignancy suppression role of miR-23a by targeting the BCR/ABL oncogene in chromic myeloid leukemia. *Cancer gene therapy* **2014,** 21, (9), 397-404.
65. Lin, R.; Zhan, M.; Yang, L.; Wang, H.; Shen, H.; Huang, S.; Huang, X.; Xu, S.; Zhang, Z.; Li, W.; Liu, Q.; Shi, Y.; Chen, W.; Yu, J.; Wang, J., Deoxycholic acid modulates the progression of gallbladder cancer through N(6)-methyladenosine-dependent microRNA maturation. *Oncogene* **2020,** 39, (26), 4983-5000.
66. Ma, H.; Wang, L. Y.; Yang, R. H.; Zhou, Y.; Zhou, P.; Kong, L., Identification of reciprocal microRNA-mRNA pairs associated with metastatic potential disparities in human prostate cancer cells and signaling pathway analysis. *Journal of cellular biochemistry* **2019,** 120, (10), 17779-17790.
67. Pal, S.; Baiocchi, R. A.; Byrd, J. C.; Grever, M. R.; Jacob, S. T.; Sif, S., Low levels of miR-92b/96 induce PRMT5 translation and H3R8/H4R3 methylation in mantle cell lymphoma. *The EMBO journal* **2007,** 26, (15), 3558-69.
68. Pan, Y.; Hu, J.; Ma, J.; Qi, X.; Zhou, H.; Miao, X.; Zheng, W.; Jia, L., MiR-193a-3p and miR-224 mediate renal cell carcinoma progression by targeting alpha-2,3-sialyltransferase IV and the phosphatidylinositol 3 kinase/Akt pathway. *Molecular carcinogenesis* **2018,** 57, (8), 1067-1077.
69. Fan, Q.; Hu, X.; Zhang, H.; Wang, S.; Zhang, H.; You, C.; Zhang, C. Y.; Liang, H.; Chen, X.; Ba, Y., MiR-193a-3p is an Important Tumour Suppressor in Lung Cancer and Directly Targets KRAS. *Cellular physiology and biochemistry : international journal of experimental cellular physiology, biochemistry, and pharmacology* **2017,** 44, (4), 1311-1324.
70. Sun, H.; Sun, Y.; Chen, Q.; Xu, Z., LncRNA KCNQ1OT1 contributes to the progression and chemoresistance in acute myeloid leukemia by modulating Tspan3 through suppressing miR-193a-3p. *Life sciences* **2020,** 241, 117161.

71. Chen, Q.; Lu, G.; Cai, Y.; Li, Y.; Xu, R.; Ke, Y.; Zhang, S., MiR-124-5p inhibits the growth of high-grade gliomas through posttranscriptional regulation of LAMB1. *Neuro-oncology* **2014,** 16, (5), 637-51.
72. von Frowein, J.; Pagel, P.; Kappler, R.; von Schweinitz, D.; Roscher, A.; Schmid, I., MicroRNA-492 is processed from the keratin 19 gene and up-regulated in metastatic hepatoblastoma. *Hepatology (Baltimore, Md.)* **2011,** 53, (3), 833-42.
73. Wang, K.; Lü, H.; Qu, H.; Xie, Q.; Sun, T.; Gan, O.; Hu, B., miR-492 Promotes Cancer Progression by Targeting GJB4 and Is a Novel Biomarker for Bladder Cancer. *OncoTargets and therapy* **2019,** 12, 11453-11464.
74. Di, Z.; Di, M.; Fu, W.; Tang, Q.; Liu, Y.; Lei, P.; Gu, X.; Liu, T.; Sun, M., Integrated Analysis Identifies a Nine-microRNA Signature Biomarker for Diagnosis and Prognosis in Colorectal Cancer. *Frontiers in genetics* **2020,** 11, 192.
75. Casabonne, D.; Benavente, Y.; Seifert, J.; Costas, L.; Armesto, M.; Arestin, M.; Besson, C.; Hosnijeh, F. S.; Duell, E. J.; Weiderpass, E.; Masala, G.; Kaaks, R.; Canzian, F.; Chirlaque, M. D.; Perduca, V.; Mancini, F. R.; Pala, V.; Trichopoulou, A.; Karakatsani, A.; La Vecchia, C.; Sánchez, M. J.; Tumino, R.; Gunter, M. J.; Amiano, P.; Panico, S.; Sacerdote, C.; Schmidt, J. A.; Boeing, H.; Schulze, M. B.; Barricarte, A.; Riboli, E.; Olsen, A.; Tjønneland, A.; Vermeulen, R.; Nieters, A.; Lawrie, C. H.; de Sanjosé, S., Serum levels of hsa-miR-16-5p, hsa-miR-29a-3p, hsa-miR-150-5p, hsa-miR-155-5p and hsa-miR-223-3p and subsequent risk of chronic lymphocytic leukemia in the EPIC study. *International journal of cancer* **2020,** 147, (5), 1315-1324.
76. Rokah, O. H.; Granot, G.; Ovcharenko, A.; Modai, S.; Pasmanik-Chor, M.; Toren, A.; Shomron, N.; Shpilberg, O., Downregulation of miR-31, miR-155, and miR-564 in chronic myeloid leukemia cells. *PloS one* **2012,** 7, (4), e35501.
77. Schneider, E.; Staffas, A.; Röhner, L.; Krowiorz, K.; Heuser, M.; Döhner, K.; Bullinger, L.; Döhner, H.; Fogelstrand, L.; Rouhi, A.; Kuchenbauer, F.; Palmqvist, L., MicroRNA-155 is upregulated in MLL-rearranged AML but its absence does not affect leukemia development. *Experimental hematology* **2016,** 44, (12), 1166-1171.
78. El-Maadawy, E. A.; Elshal, M. F.; Bakry, R. M.; Moussa, M. M.; El-Naby, S.; Talaat, R. M., Regulation of CD4(+)CD25(+)FOXP3(+) cells in Pediatric Acute Lymphoblastic Leukemia (ALL): Implication of cytokines and miRNAs. *Molecular immunology* **2020,** 124, 1-8.
79. Ferrajoli, A.; Shanafelt, T. D.; Ivan, C.; Shimizu, M.; Rabe, K. G.; Nouraee, N.; Ikuo, M.; Ghosh, A. K.; Lerner, S.; Rassenti, L. Z.; Xiao, L.; Hu, J.; Reuben, J. M.; Calin, S.; You, M. J.; Manning, J. T.; Wierda, W. G.; Estrov, Z.; O'Brien, S.; Kipps, T. J.; Keating, M. J.; Kay, N. E.; Calin, G. A., Prognostic value of miR-155 in individuals with monoclonal B-cell lymphocytosis and patients with B chronic lymphocytic leukemia. *Blood* **2013,** 122, (11), 1891-9.
80. Fulci, V.; Chiaretti, S.; Goldoni, M.; Azzalin, G.; Carucci, N.; Tavolaro, S.; Castellano, L.; Magrelli, A.; Citarella, F.; Messina, M.; Maggio, R.; Peragine, N.; Santangelo, S.; Mauro, F. R.; Landgraf, P.; Tuschl, T.; Weir, D. B.; Chien, M.; Russo, J. J.; Ju, J.; Sheridan, R.; Sander, C.; Zavolan, M.; Guarini, A.; Foà, R.; Macino, G., Quantitative technologies establish a novel microRNA profile of chronic lymphocytic leukemia. *Blood* **2007,** 109, (11), 4944-51.
81. Han, B.; Wang, S.; Zhao, H., MicroRNA-21 and microRNA-155 promote the progression of Burkitt's lymphoma by the PI3K/AKT signaling pathway. *International journal of clinical and experimental pathology* **2020,** 13, (1), 89-98.
82. Borchert, G. M.; Holton, N. W.; Larson, E. D., Repression of human activation induced cytidine deaminase by miR-93 and miR-155. *BMC cancer* **2011,** 11, 347.
83. Kluiver, J.; Haralambieva, E.; de Jong, D.; Blokzijl, T.; Jacobs, S.; Kroesen, B. J.; Poppema, S.; van den Berg, A., Lack of BIC and microRNA miR-155 expression in

primary cases of Burkitt lymphoma. *Genes, chromosomes & cancer* **2006,** 45, (2), 147-53.
84. Lin, X.; Wang, Z.; Zhang, R.; Feng, W., High serum microRNA-335 level predicts aggressive tumor progression and unfavorable prognosis in pediatric acute myeloid leukemia. *Clinical & translational oncology : official publication of the Federation of Spanish Oncology Societies and of the National Cancer Institute of Mexico* **2015,** 17, (5), 358-64.
85. Ronchetti, D.; Lionetti, M.; Mosca, L.; Agnelli, L.; Andronache, A.; Fabris, S.; Deliliers, G. L.; Neri, A., An integrative genomic approach reveals coordinated expression of intronic miR-335, miR-342, and miR-561 with deregulated host genes in multiple myeloma. *BMC medical genomics* **2008,** 1, 37.
86. Saleh, L. M.; Wang, W.; Herman, S. E.; Saba, N. S.; Anastas, V.; Barber, E.; Corrigan-Cummins, M.; Farooqui, M.; Sun, C.; Sarasua, S. M.; Zhao, Z.; Abousamra, N. K.; Elbaz, O.; Abdelghaffar, H. A.; Wiestner, A.; Calvo, K. R., Ibrutinib downregulates a subset of miRNA leading to upregulation of tumor suppressors and inhibition of cell proliferation in chronic lymphocytic leukemia. *Leukemia* **2017,** 31, (2), 340-349.
87. Saccomani, V.; Grassi, A.; Piovan, E.; Bongiovanni, D.; Di Martino, L.; Minuzzo, S.; Tosello, V.; Zanovello, P., miR-22-3p Negatively Affects Tumor Progression in T-Cell Acute Lymphoblastic Leukemia. *Cells* **2020,** 9, (7).
88. Qu, H.; Zheng, G.; Cheng, S.; Xie, W.; Liu, X.; Tao, Y.; Xie, B., Serum miR-22 is a novel prognostic marker for acute myeloid leukemia. *Journal of clinical laboratory analysis* **2020**, e23370.
89. Wei, D.; Miao, Y.; Yu, L.; Wang, D.; Wang, Y., Downregulation of microRNA-198 suppresses cell proliferation and invasion in retinoblastoma by directly targeting PTEN. *Molecular medicine reports* **2018,** 18, (1), 595-602.
90. Marin-Muller, C.; Li, D.; Bharadwaj, U.; Li, M.; Chen, C.; Hodges, S. E.; Fisher, W. E.; Mo, Q.; Hung, M. C.; Yao, Q., A tumorigenic factor interactome connected through tumor suppressor microRNA-198 in human pancreatic cancer. *Clinical cancer research : an official journal of the American Association for Cancer Research* **2013,** 19, (21), 5901-13.
91. Wang, S.; Zhang, X.; Yang, C.; Xu, S., MicroRNA-198-5p inhibits the migration and invasion of non-small lung cancer cells by targeting fucosyltransferase 8. *Clinical and experimental pharmacology & physiology* **2019,** 46, (10), 955-967.
92. Zhu, G.; Wang, Z.; Mijiti, M.; Du, G.; Li, Y.; Dangmurenjiafu, G., MiR-28-5p promotes human glioblastoma cell growth through inactivation of FOXO1. *International journal of clinical and experimental pathology* **2019,** 12, (8), 2972-2980.
93. Xiao, F.; Cheng, Z.; Wang, P.; Gong, B.; Huang, H.; Xing, Y.; Liu, F., MicroRNA-28-5p inhibits the migration and invasion of gastric cancer cells by suppressing AKT phosphorylation. *Oncology letters* **2018,** 15, (6), 9777-9785.
94. Bartolomé-Izquierdo, N.; de Yébenes, V. G.; Álvarez-Prado, A. F.; Mur, S. M.; Lopez Del Olmo, J. A.; Roa, S.; Vazquez, J.; Ramiro, A. R., miR-28 regulates the germinal center reaction and blocks tumor growth in preclinical models of non-Hodgkin lymphoma. *Blood* **2017,** 129, (17), 2408-2419.
95. Schneider, C.; Setty, M.; Holmes, A. B.; Maute, R. L.; Leslie, C. S.; Mussolin, L.; Rosolen, A.; Dalla-Favera, R.; Basso, K., MicroRNA 28 controls cell proliferation and is down-regulated in B-cell lymphomas. *Proceedings of the National Academy of Sciences of the United States of America* **2014,** 111, (22), 8185-90.
96. Wang, X. X.; Zhang, H.; Li, Y., Preliminary study on the role of miR-148a and DNMT1 in the pathogenesis of acute myeloid leukemia. *Molecular medicine reports* **2019,** 19, (4), 2943-2952.

97. Li, X.; Zou, W.; Wang, Y.; Liao, Z.; Li, L.; Zhai, Y.; Zhang, L.; Gu, S.; Zhao, X., Plasma-based microRNA signatures in early diagnosis of breast cancer. *Molecular genetics & genomic medicine* **2020,** 8, (5), e1092.
98. Bhayadia, R.; Krowiorz, K.; Haetscher, N.; Jammal, R.; Emmrich, S.; Obulkasim, A.; Fiedler, J.; Schwarzer, A.; Rouhi, A.; Heuser, M.; Wingert, S.; Bothur, S.; Döhner, K.; Mätzig, T.; Ng, M.; Reinhardt, D.; Döhner, H.; Zwaan, C. M.; van den Heuvel Eibrink, M.; Heckl, D.; Fornerod, M.; Thum, T.; Humphries, R. K.; Rieger, M. A.; Kuchenbauer, F.; Klusmann, J. H., Endogenous Tumor Suppressor microRNA-193b: Therapeutic and Prognostic Value in Acute Myeloid Leukemia. *Journal of clinical oncology : official journal of the American Society of Clinical Oncology* **2018,** 36, (10), 1007-1016.
99. Chen, J.; Zhang, X.; Lentz, C.; Abi-Daoud, M.; Paré, G. C.; Yang, X.; Feilotter, H. E.; Tron, V. A., miR-193b Regulates Mcl-1 in Melanoma. *The American journal of pathology* **2011,** 179, (5), 2162-8.
100. Raychaudhuri, M.; Bronger, H.; Buchner, T.; Kiechle, M.; Weichert, W.; Avril, S., MicroRNAs miR-7 and miR-340 predict response to neoadjuvant chemotherapy in breast cancer. *Breast cancer research and treatment* **2017,** 162, (3), 511-521.
101. Yu, Z.; Ni, L.; Chen, D.; Zhang, Q.; Su, Z.; Wang, Y.; Yu, W.; Wu, X.; Ye, J.; Yang, S.; Lai, Y.; Li, X., Identification of miR-7 as an oncogene in renal cell carcinoma. *Journal of molecular histology* **2013,** 44, (6), 669-77.
102. Sun, H.; Zhang, Z.; Luo, W.; Liu, J.; Lou, Y.; Xia, S., MiR-7 Functions as a Tumor Suppressor by Targeting the Oncogenes TAL1 in T-Cell Acute Lymphoblastic Leukemia. *Technology in cancer research & treatment* **2020,** 19, 1533033820934130.
103. Xin, L.; Liu, L.; Liu, C.; Zhou, L. Q.; Zhou, Q.; Yuan, Y. W.; Li, S. H.; Zhang, H. T., DNA-methylation-mediated silencing of miR-7-5p promotes gastric cancer stem cell invasion via increasing Smo and Hes1. *Journal of cellular physiology* **2020,** 235, (3), 2643-2654.
104. Organista-Nava, J.; Gómez-Gómez, Y.; Illades-Aguiar, B.; Del Carmen Alarcón-Romero, L.; Saavedra-Herrera, M. V.; Rivera-Ramírez, A. B.; Garzón-Barrientos, V. H.; Leyva-Vázquez, M. A., High miR-24 expression is associated with risk of relapse and poor survival in acute leukemia. *Oncology reports* **2015,** 33, (4), 1639-49.
105. Xu, D. F.; Wang, L. S.; Zhou, J. H., Long non-coding RNA CASC2 suppresses pancreatic cancer cell growth and progression by regulating the miR-24/MUC6 axis. *International journal of oncology* **2020,** 56, (2), 494-507.
106. Jiang, W.; Meng, K.; Sheng, G.; Yang, T., MicroRNA-24 inhibits the proliferation, migration and invasion and enhances chemosensitivity of human gastric cancer by targeting DND1. *Journal of B.U.ON. : official journal of the Balkan Union of Oncology* **2020,** 25, (2), 1001-1006.
107. Wang, F.; Ye, B. G.; Liu, J. Z.; Kong, D. L., miR-487b and TRAK2 that form an axis to regulate the aggressiveness of osteosarcoma, are potential therapeutic targets and prognostic biomarkers. *Journal of biochemical and molecular toxicology* **2020**, e22511.
108. Chen, X.; Lin, Z. F.; Xi, W. J.; Wang, W.; Zhang, D.; Yang, F.; Li, Y. F.; Huo, Y.; Zhang, T. Z.; Jiang, Y. H.; Qin, W. W.; Yang, A. G.; Wang, T., DNA methylation-regulated and tumor-suppressive roles of miR-487b in colorectal cancer via targeting MYC, SUZ12, and KRAS. *Cancer medicine* **2019,** 8, (4), 1694-1709.
109. Gattolliat, C. H.; Thomas, L.; Ciafrè, S. A.; Meurice, G.; Le Teuff, G.; Job, B.; Richon, C.; Combaret, V.; Dessen, P.; Valteau-Couanet, D.; May, E.; Busson, P.; Douc-Rasy, S.; Bénard, J., Expression of miR-487b and miR-410 encoded by 14q32.31 locus is a prognostic marker in neuroblastoma. *British journal of cancer* **2011,** 105, (9), 1352-61.
110. Hua, K. T.; Hong, J. B.; Sheen, Y. S.; Huang, H. Y.; Huang, Y. L.; Chen, J. S.; Liao, Y. H., miR-519d Promotes Melanoma Progression by Downregulating EphA4. *Cancer research* **2018,** 78, (1), 216-229.

111. Xie, Q.; Wang, S.; Zhao, Y.; Zhang, Z.; Qin, C.; Yang, X., MiR-519d impedes cisplatin-resistance in breast cancer stem cells by down-regulating the expression of MCL-1. *Oncotarget* **2017,** 8, (13), 22003-22013.
112. Xu, Z.; Li, Z.; Wang, W.; Xia, Y.; He, Z.; Li, B.; Wang, S.; Huang, X.; Sun, G.; Xu, J.; Wang, L.; Zhang, Q.; Li, Q.; Lv, J.; Wang, L.; Zhang, L.; Zhang, D.; Xu, H.; Xu, Z., MIR-1265 regulates cellular proliferation and apoptosis by targeting calcium binding protein 39 in gastric cancer and, thereby, impairing oncogenic autophagy. *Cancer letters* **2019,** 449, 226-236.
113. Luo, M.; Zhang, Q.; Xia, M.; Hu, F.; Ma, Z.; Chen, Z.; Guo, A. Y., Differential Co-expression and Regulatory Network Analysis Uncover the Relapse Factor and Mechanism of T Cell Acute Leukemia. *Molecular therapy. Nucleic acids* **2018,** 12, 184-194.
114. Wei, J.; Yang, L.; Wu, Y. N.; Xu, J., Serum miR-1290 and miR-1246 as Potential Diagnostic Biomarkers of Human Pancreatic Cancer. *Journal of Cancer* **2020,** 11, (6), 1325-1333.
115. Rosato, P.; Anastasiadou, E.; Garg, N.; Lenze, D.; Boccellato, F.; Vincenti, S.; Severa, M.; Coccia, E. M.; Bigi, R.; Cirone, M.; Ferretti, E.; Campese, A. F.; Hummel, M.; Frati, L.; Presutti, C.; Faggioni, A.; Trivedi, P., Differential regulation of miR-21 and miR-146a by Epstein-Barr virus-encoded EBNA2. *Leukemia* **2012,** 26, (11), 2343-52.
116. Chen, G.; Chen, Z.; Zhao, H., MicroRNA-155-3p promotes glioma progression and temozolomide resistance by targeting Six1. *Journal of cellular and molecular medicine* **2020,** 24, (9), 5363-5374.
117. Zhang, G.; Zhong, L.; Luo, H.; Wang, S., MicroRNA-155-3p promotes breast cancer progression through down-regulating CADM1. *OncoTargets and therapy* **2019,** 12, 7993-8002.
118. Tang, B.; Lei, B.; Qi, G.; Liang, X.; Tang, F.; Yuan, S.; Wang, Z.; Yu, S.; He, S., MicroRNA-155-3p promotes hepatocellular carcinoma formation by suppressing FBXW7 expression. *Journal of experimental & clinical cancer research : CR* **2016,** 35, (1), 93.
119. Yim, R. L.; Wong, K. Y.; Kwong, Y. L.; Loong, F.; Leung, C. Y.; Chu, R.; Lam, W. W.; Hui, P. K.; Lai, R.; Chim, C. S., Methylation of miR-155-3p in mantle cell lymphoma and other non-Hodgkin's lymphomas. *Oncotarget* **2014,** 5, (20), 9770-82.
120. Gimenes-Teixeira, H. L.; Lucena-Araujo, A. R.; Dos Santos, G. A.; Zanette, D. L.; Scheucher, P. S.; Oliveira, L. C.; Dalmazzo, L. F.; Silva-Júnior, W. A.; Falcão, R. P.; Rego, E. M., Increased expression of miR-221 is associated with shorter overall survival in T-cell acute lymphoid leukemia. *Experimental hematology & oncology* **2013,** 2, (1), 10.
121. Cammarata, G.; Augugliaro, L.; Salemi, D.; Agueli, C.; La Rosa, M.; Dagnino, L.; Civiletto, G.; Messana, F.; Marfia, A.; Bica, M. G.; Cascio, L.; Floridia, P. M.; Mineo, A. M.; Russo, M.; Fabbiano, F.; Santoro, A., Differential expression of specific microRNA and their targets in acute myeloid leukemia. *American journal of hematology* **2010,** 85, (5), 331-9.
122. Zhang, L.; Li, H.; Yuan, M.; Li, M.; Zhang, S., Cervical Cancer Cells-Secreted Exosomal microRNA-221-3p Promotes Invasion, Migration and Angiogenesis of Microvascular Endothelial Cells in Cervical Cancer by Down-Regulating MAPK10 Expression. *Cancer management and research* **2019,** 11, 10307-10319.
123. Felli, N.; Fontana, L.; Pelosi, E.; Botta, R.; Bonci, D.; Facchiano, F.; Liuzzi, F.; Lulli, V.; Morsilli, O.; Santoro, S.; Valtieri, M.; Calin, G. A.; Liu, C. G.; Sorrentino, A.; Croce, C. M.; Peschle, C., MicroRNAs 221 and 222 inhibit normal erythropoiesis and erythroleukemic cell growth via kit receptor down-modulation. *Proceedings of the*

National Academy of Sciences of the United States of America **2005,** 102, (50), 18081-6.

124. Frenquelli, M.; Muzio, M.; Scielzo, C.; Fazi, C.; Scarfò, L.; Rossi, C.; Ferrari, G.; Ghia, P.; Caligaris-Cappio, F., MicroRNA and proliferation control in chronic lymphocytic leukemia: functional relationship between miR-221/222 cluster and p27. *Blood* **2010,** 115, (19), 3949-59.
125. Song, Q.; An, Q.; Niu, B.; Lu, X.; Zhang, N.; Cao, X., Role of miR-221/222 in Tumor Development and the Underlying Mechanism. *Journal of oncology* **2019,** 2019, 7252013.
126. Gao, R.; Wang, Z.; Liu, Q.; Yang, C., MicroRNA-105 plays an independent prognostic role in esophageal cancer and acts as an oncogene. *Cancer biomarkers : section A of Disease markers* **2020,** 27, (2), 173-180.
127. Shang, J. C.; Yu, G. Z.; Ji, Z. W.; Wang, X. Q.; Xia, L., MiR-105 inhibits gastric cancer cells metastasis, epithelial-mesenchymal transition by targeting SOX9. *European review for medical and pharmacological sciences* **2019,** 23, (14), 6160-6169.
128. Li, J.; Zhang, Z.; Chen, F.; Hu, T.; Peng, W.; Gu, Q.; Sun, Y., The Diverse Oncogenic and Tumor Suppressor Roles of microRNA-105 in Cancer. *Frontiers in oncology* **2019,** 9, 518.
129. Pekarsky, Y.; Croce, C. M., Role of miR-15/16 in CLL. *Cell death and differentiation* **2015,** 22, (1), 6-11.
130. Lovat, F.; Nigita, G.; Distefano, R.; Nakamura, T.; Gasparini, P.; Tomasello, L.; Fadda, P.; Ibrahimova, N.; Catricalà, S.; Palamarchuk, A.; Caligiuri, M. A.; Gallì, A.; Malcovati, L.; Minden, M. D.; Croce, C. M., Combined loss of function of two different loci of miR-15/16 drives the pathogenesis of acute myeloid leukemia. *Proceedings of the National Academy of Sciences of the United States of America* **2020,** 117, (22), 12332-12340.
131. Calin, G. A.; Dumitru, C. D.; Shimizu, M.; Bichi, R.; Zupo, S.; Noch, E.; Aldler, H.; Rattan, S.; Keating, M.; Rai, K.; Rassenti, L.; Kipps, T.; Negrini, M.; Bullrich, F.; Croce, C. M., Frequent deletions and down-regulation of micro- RNA genes miR15 and miR16 at 13q14 in chronic lymphocytic leukemia. *Proceedings of the National Academy of Sciences of the United States of America* **2002,** 99, (24), 15524-9.
132. Zhang, X.; Chen, X.; Lin, J.; Lwin, T.; Wright, G.; Moscinski, L. C.; Dalton, W. S.; Seto, E.; Wright, K.; Sotomayor, E.; Tao, J., Myc represses miR-15a/miR-16-1 expression through recruitment of HDAC3 in mantle cell and other non-Hodgkin B-cell lymphomas. *Oncogene* **2012,** 31, (24), 3002-3008.
